# SPACESHIP: Autonomous Mapping of Hardware-Dependent Synthesizable Space in Solution-Phase Gold Nanomaterials

**DOI:** 10.1021/jacs.6c03132

**Published:** 2026-04-29

**Authors:** Nayeon Kim, Hyuk Jun Yoo, Daeho Kim, Heeseung Lee, Chang Seop Hong, Sang Soo Han

**Affiliations:** † Computational Science Research Center, 58975 Korea Institute of Science and Technology, Seoul 02792, Republic of Korea; ‡ Department of Chemistry, Korea University, Seoul 02841, Republic of Korea; § Department of Chemical and Biological Engineering, Korea University, Seoul 02841, Republic of Korea; ∥ Department of Materials Science and Engineering, Korea University, Seoul 02841, Republic of Korea

## Abstract

Autonomous laboratories hold great promise for accelerating materials discovery but often inherit hidden limits because experimental boundaries have been predefined by human intuition or literature precedents. Such a priori constraints risk excluding feasible regions, particularly since synthesizable conditions can shift with hardware or environmental factors. We present SPACESHIP, an AI framework integrated with automated experimental hardware for adaptive exploration of chemical spaces free from literature- or expert-derived feasibility constraints. Through an AI-based prediction, robotic synthesis, real-time characterization, and model update, SPACESHIP combines probabilistic models with an *Autopilot* acquisition strategy that dynamically switches between models to refine synthesizable regions using both successful and failed experiments. Applied to gold nanoparticle (NP) and nanorod (NR) synthesis, this AI–robotics system achieved 90% accuracy in only 23 experiments, compared with 512 required for the ground truth. It uncovered distinct growth regimes across optical property classes and expanded synthesizable regions by factors of 8 (NPs) and 4 (NRs) beyond literature maps, adapting to hardware-specific conditions rather than relying on fixed, external constraints. By merging machine learning with autonomous experimentation, SPACESHIP addresses the long-standing reproducibility gap in science by diagnosing and adapting to system-specific synthesizable boundaries that shift across laboratories and environments, rather than assuming one universal map.

## Graphical abstract

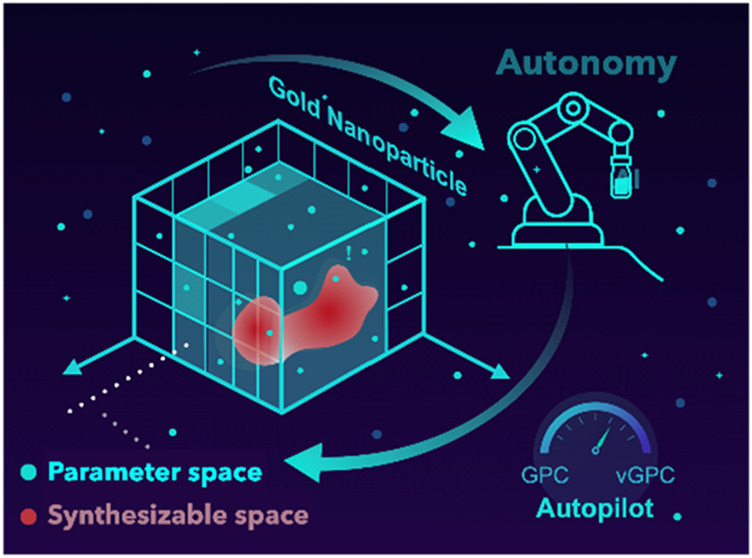

## Introduction

Autonomous laboratories that integrate robotics with AI-driven decision-making, have emerged as powerful tools for accelerating discovery in chemistry,^1,2^ materials science,^3–5^ and biotechnology.^6,7^ By automating the full experimental loop, including design,^8–10^ execution,^11–13^ and analysis,^14,15^ these systems enable rapid, reproducible exploration of complex parameter spaces. In most current implementations, however, the AI component is primarily tasked with optimization^16–18^ selecting experimental conditions that maximize a predefined objective. This process is typically carried out within a fixed set of constraints,^19,20^ which are established in advance by researchers on the basis of heuristics or prior experience. While effective for searching within specific domains, such systems implicitly assume that the relevant design space has been correctly defined from the outset.

Despite their automated appearance, current autonomous laboratories remain fundamentally limited by these human-imposed boundaries,^21^ which are rarely challenged or updated during operation. In the absence of a systematic alternative, researchers often rely on empirical approximations to guide optimization. This trial-and-error approach frequently leads to a mischaracterized experimental space and several recurring inefficiencies.^22,23^ First, sampling physically infeasible regions wastes experimental resources and generates noninformative data, which in turn degrade the performance of AI models for autonomous laboratories.^24,25^ Second, near-success conditions, such as results with marginally undetectable concentrations, are often misclassified as complete failures, limiting the model’s capacity to learn from partial successes.^26,27^ Third, overly narrow constraints can hinder exploration, increasing the risk of overlooking optimal regions or converging to suboptimal solutions. These issues are rarely detected and corrected during the course of experimentation.^28,29^ More fundamentally, relying on human intuition to define the synthesizable space in materials discovery is inherently time-consuming and prone to error, regardless of whether or not such errors are eventually identified.^30,31^ While human researchers often vary only a few parameters at a time, holding others constant for practical control, AI-driven platforms can simultaneously explore dozens of variables. As a result, defining the synthesizable space becomes far more challenging and emerges as a critical prerequisite for autonomous discovery, rather than an afterthought.

This challenge is exacerbated by the high sensitivity of material synthesis to minor changes in experimental parameters, which can alter the synthesizable space.^32,33^ These shifts often render previously defined constraints invalid, requiring a complete reset of the search process and necessitating human intervention, directly contracting the core purpose of autonomous laboratories. This contradiction becomes even more pronounced in high-dimensional experimental spaces, where the complexity of variable interactions exceeds the capacity of human intuition to effectively guide exploration.^34^ To address these limitations, it is essential to develop AI models capable of autonomously inferring and adapting their experimental boundaries in response to evolving conditions.

In this study, we introduce an AI model named **SPACESHIP** (**S**ynthesizable **P**arameter **A**cquisition via **C**losed-loop **E**xploration using **S**elf-directed, **H**ardware-aware **I**ntelligent **P**rotocols), which is designed to define synthesizable regions for materials synthesis, with direct applicability to autonomous laboratories. The model incorporates an adaptive mechanism, which we term *Autopilot*, that dynamically adjusts its exploration strategy based on the complexity of the design space and the volume of available data. This enables efficient and flexible navigation of experimental spaces without the need for predefined constraints or manual intervention. We demonstrate the practical effectiveness of this approach through the synthesis of gold (Au) nanomaterials. First, the model is applied to a binary classification task inspired by the classical Turkevich method,^35^ where the objective is to predict whether a given condition results in successful nanoparticle (NP) formation. We then extend the approach to a multiclass^36–38^ classification setting via the seed-mediated growth of Au nanorods (NRs). In this context, the model classifies outcomes not only by synthesis success but also by the achievement of specific target shapes or optical properties. Supported by these experimental validations, we show that an autonomous laboratory equipped with our model can independently update its understanding of the synthesizable space as conditions change. This allows for the targeting of desired property classes without relying on human-defined constraints, making a significant step toward a fully autonomous and scalable platform for materials discovery.

## Results and Discussion

### Terminology Definitions

To define the synthesizable space that enables autonomous learning of experimental boundaries and facilitates multitarget space exploration, we begin by introducing key terminologies that underpin the autonomous exploration framework in this study: the *parameter space*, the *synthesizable space*, and the *optimal space*. These concepts, illustrated in Figure 1a, form the basis for understanding how the system identifies feasible experimental conditions and iteratively optimizes toward desired targets without relying on human-defined feasibility constraints based on literature precedents.

**Figure 1:**
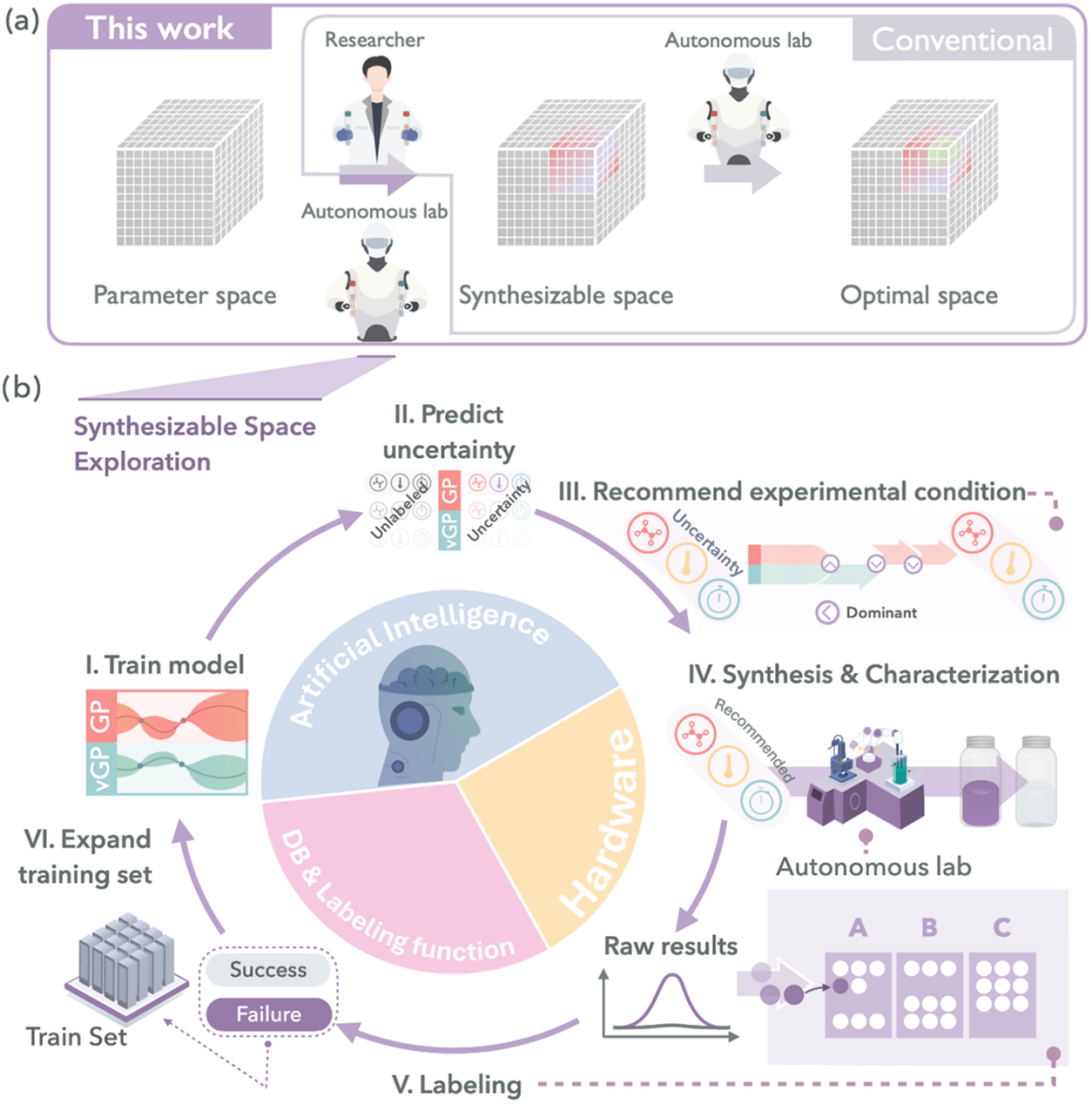
Conceptual framework and autonomous experimental workflow for mapping synthesizable space. (a) The parameter space includes all physically executable combinations defined by the experimental hardware. The synthesizable space is a subset where synthesis is successful and further refined into target-specific regions. The optimization is performed within this space to identify the desired outcomes. (b) Workflow of autonomous synthesizable space exploration via active learning. (I) Probabilistic models (GPC and vGPC) are initially trained on existing labeled data. (II) These models estimate the predictive uncertainty for unlabeled experimental conditions. (III) An acquisition function uses this uncertainty to select the next most informative experimental condition. (IV) The autonomous laboratory platform executes the selected experimental conditions and records the corresponding measurement data. (V) Experimental outcomes are automatically labeled according to predefined criteria. (VI) The newly labeled data are incorporated into the training set, enabling iterative refinement of the model’s predictive accuracy and further optimization of the experimental search.

The parameter space refers to the complete set of experimental variables that are theoretically accessible within the physical and operational limits of the experimental hardware. This space encompasses all combinations of conditions that can be executed by the robotic platform in an autonomous laboratory, regardless of whether those conditions ultimately succeed or fail in achieving material synthesis. In constructing this space, we integrate knowledge of feasible experimental settings with hardware-specific constraints to generate conditional vectors. For example, information such as chemical identity and pump configuration can be combined and encoded into a conditional vector specifying parameters such as concentration, dispensing volume, and flow rate for each reagent. This represents one approach to construct the parameter space; the exact formulation may vary depending on the specific experimental setup. The full parameter space is then defined by the combinatorial expansion of all such conditional vectors. A schematic overview of this process is provided in Figure S1, which illustrates how chemical and hardware parameters are translated into conditional vectors that define executable experimental conditions.

The synthesizable space is a subset of the parameter space, representing the region where successful synthesis is experimentally detectable. This space can be categorized into two distinct types. The first is the basic synthesizable space, defined by experimental conditions that yield measurable signals detectable by the system’s instrumentation. This effectively establishes a “hard boundary” for what is experimentally feasible. The second is the targeted synthesizable space, a refined subset of the basic space that encompasses conditions producing specific, desirable outcomes such as characteristic morphologies, functional surface properties, distinctive spectral signatures, or catalytic behaviors. This distinction allows not only the detection of any synthesis event but also the precise classification of outcomes on the basis of predefined performance or property criteria.

Crucially, distinguishing between the synthesizable space and the optimal space is essential for efficient autonomous experimentation. Attempting to directly optimize across the entire parameter space without first identifying regions where synthesis is feasible can lead to excessive trial-and-error and high failure rates, particularly in high-dimensional or partially unknown systems. Preemptively identifying feasibility boundaries prior to optimization provides a strategic advantage, particularly when evaluating constraints is less resource-intensive or yields more informative feedback than objective evaluations do. By first learning the boundaries of the synthesizable space, optimization can be confined to feasible regions, enabling the system to allocate resources more efficiently and focus exploration on meaningful subspaces within the broader parameter domain.

Once the synthesizable space is established, optimization algorithms used in autonomous experimentation can be applied to identify the optimal conditions, those that maximize performance relative to a specific target metric. Unlike traditional workflows, which rely on expert-defined feasibility regions or heuristic assumptions, the methodology introduced in this study empowers autonomous laboratories to dynamically learn and update their own synthesizable space in response to real-time experimental feedback. This capability removes the bottleneck of manual constraint definition and allows our framework to adaptively refine its search boundaries, even in complex, high-dimensional, and evolving environments. Ultimately, this approach marks a significant step toward fully autonomous, intelligent experimentation.

### Closed-Loop Workflow and Model Architecture of SPACESHIP

Determining whether a given synthesis condition is synthesizable is formulated as a classification problem. Within this framework, AI models operating in an autonomous laboratory are trained to predict whether a specific set of input parameters falls within the synthesizable region of the experimental design space. To address this challenge, SPACESHIP was developed on the basis of an active learning framework.^39^ By prioritizing conditions associated with high predictive uncertainty or great potential to improve model performance, SPACESHIP enables targeted data acquisition that enhances its understanding of the synthesizable space.

Figure 1b illustrates a closed-loop workflow of SPACESHIP, which integrates probabilistic modeling with autonomous experimentation. The process comprises six sequential steps: (I) training a classifier using existing data, (II) estimating predictive uncertainty for unexplored synthesis conditions, (III) selecting the next experimental condition using an acquisition function, (IV) executing the experiment via a robotic platform, (V) labeling the experimental outcome according to predefined criteria, and (VI) updating the model with the newly acquired data. Repeating this cyclic workflow allows autonomous laboratories to iteratively refine model accuracy and progressively map the boundaries of the synthesizable region with increasing precision. A formal description of this iterative procedure is provided in Algorithm S1.

At the core of SPACESHIP lies its acquisition strategy (step III), which determines the selection of subsequent experimental conditions. This strategy employs two probabilistic models, Gaussian process classifiers (GPCs)^40^ and variational GPC (vGPC),^41^ to estimate the probability of synthesizability for candidate conditions while simultaneously quantifying the associated predictive uncertainty.^42^ This adaptive acquisition function, referred to as *Autopilot*, dynamically guides experimental selection by accounting for data sparsity and task complexity within the design space. The details of *Autopilot* are provided in the Experimental Section. The condition with the highest acquisition score is executed autonomously by the robotic system, and the outcome, including both success and failure data, is automatically labeled. This newly obtained data point is then incorporated into the training set, thereby closing the loop and enabling continuous refinement of the predictive model.

We evaluate how the model architecture and acquisition strategy influence the efficiency of the synthesizable space search within the closed-loop system introduced in Figure 1b. Benchmarking was conducted using the Olympus simulation platform,^43^ which offers a scalable and cost-effective environment for validating learning strategies through virtual simulation tests prior to laboratory execution. For this purpose, we used the HyperEllipsoid surface (Figure S2), a smooth, convex optimization landscape chosen for its topological resemblance to experimental design spaces, where synthesizable regions are typically continuous and exhibit gradual transitions. To enable classification-based evaluation, a threshold-based labeling scheme was applied to the continuous output, as detailed in Note S1. Additional simulations using the Rosenbrock and Dejong surfaces, featuring narrow ridge-like structures and island-shaped regions, respectively, were included to assess model robustness across structurally diverse search spaces.

The synthesizability prediction task was formalized as a binary classification problem aimed at identifying whether a given condition satisfies constraints for synthesis feasibility. In Figure 2a, model performance is compared across three data acquisition strategies: random sampling, confidence-based active learning (typical active learning), and acquisition function-driven active learning (*suggested in this work*). For each setting, we measured the proportion of the parameter space that needed to be sampled before reaching 90% predictive accuracy, which served as a practical metric for learning efficiency. The initial comparison under random sampling revealed that nonprobabilistic models such as XGBoost^44^ require substantially more samples to reach the target performance.^65^ TabPFN, while demonstrating improved performance compared to tree-based methods, still exhibited limited sample efficiency and large variance in this low-data regime. To further assess the role of probabilistic modeling, we additionally evaluated probabilistic approaches, including Gaussian Process Ensemble (GPE)^66^ and Gaussian Process Classifiers (GPC and vGPC). GPE showed improved performance compared to nonprobabilistic baselines such as XGBoost, but did not outperform TabPFN under the same conditions. Although ensemble-based uncertainty estimation is widely used, it may lack sufficient resolution for effective active learning in extremely low-data regimes, which likely limited the performance of GPE in this study. In contrast, GPC- and vGPC-based models consistently demonstrated superior sample efficiency and the most robust performance across repeated trials. Additional baseline models were evaluated but exhibited lower performance; detailed results are provided in Note S2 and the benchmark model settings are mentioned in Notes S1–S3. On the basis of these observations, we excluded nonprobabilistic and intermediate-performing models from further analysis and focused on GPCs and vGPC.

**Figure 2:**
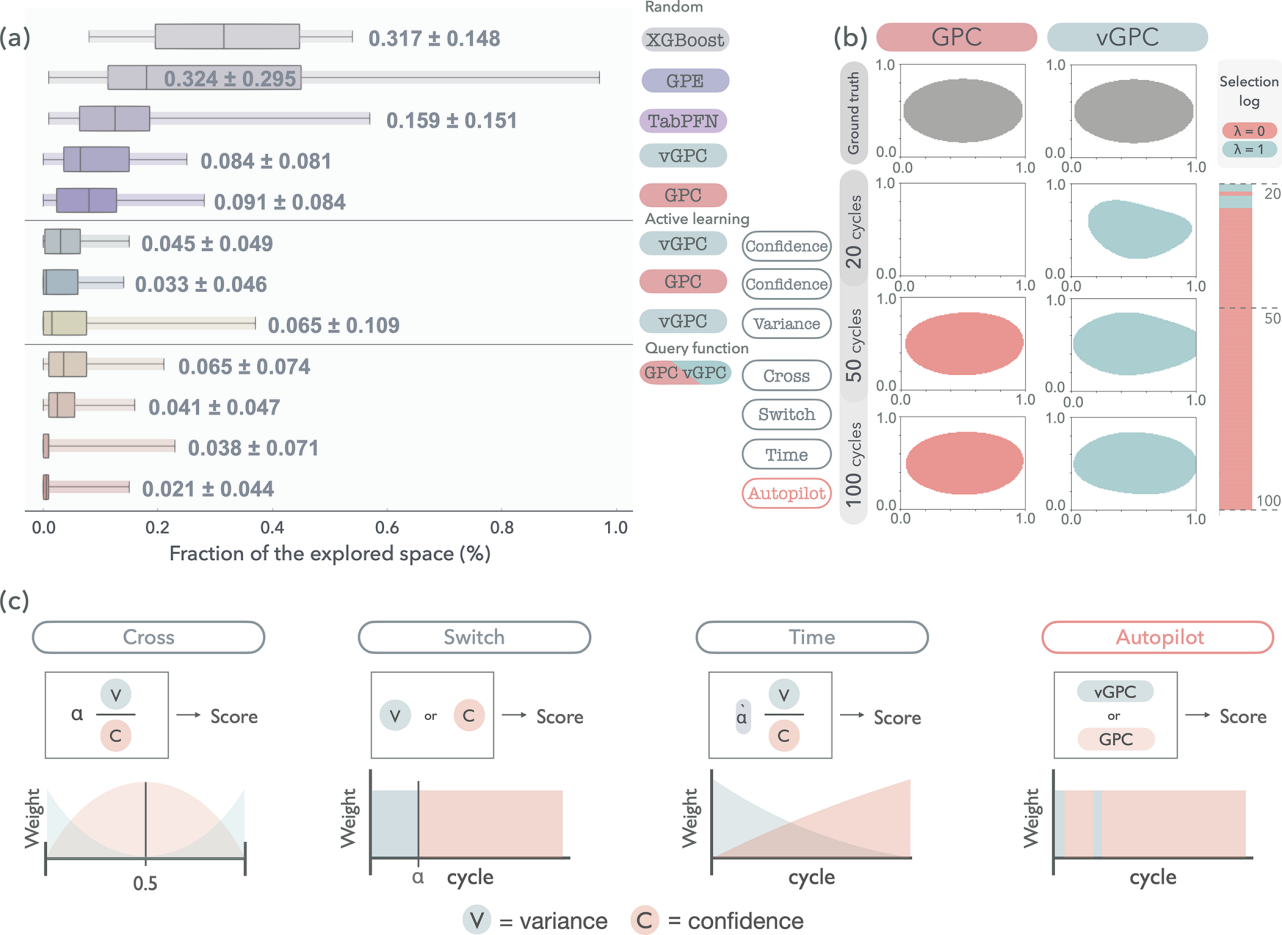
Performance comparison of exploration strategies and model behavior in virtual experiments. (a) Search efficiency in identifying the synthesizable space across different models and acquisition functions. Boxes indicate the interquartile range with the median marked inside, while error bars show standard deviation over multiple runs. (b) Evolution of the model’s decision boundary as a function of increasing the numbers of cycles. (c) Schematic illustrations and definitions of the acquisition functions: *Cross*, *Switch*, *Time*, and *Autopilot*.

Even when paired with a simple confidence-based acquisition strategy, both models substantially outperform random sampling. To advance beyond conventional active learning techniques, we developed four acquisition strategies, *Cross*, *Switch*, *Time*, and *Autopilot*. Detailed mathematical formulations of these strategies are provided in the Experimental Section, and the evolution of the synthesizability decision boundary, reflecting the model’s learning progress, is depicted inFigure [Fig fig2]b. Each strategy defined the synthesizable space more efficiently than did a random search. Specifically, *Cross* required 1.4 times fewer acquisition cycles (1 cycle = 1 data), whereas *Switch* and *Time* required 2.2 and 2.3 times fewer acquisition cycles, and *Autopilot* achieved a 4.3-fold acceleration (Figure 2a).

Notably, *Autopilot* achieved the highest efficiency by dynamically selecting between the GPC and vGPC during each acquisition cycle on the basis of real-time validation performance. While both surrogate models benefit from confidence-based sampling, their predictive behavior diverges depending on the quantity and complexity of the available data. To gain insight into uncertainty-driven model behavior, we first applied our framework to benchmark tasks with simple decision surfaces like those in general experimental settings and visualized how the decision boundaries evolved as labeled data accumulated (Figure 2c). We confirmed that these effects are strongly influenced by the underlying data complexity. As shown in Supporting Figures S3–S6, the GPC performed best on sufficient data sets or those with low intrinsic complexity, conditions under which the decision surface was relatively smooth and could be approximated with fewer parameters. In contrast, vGPC outperformed GPC in data-scarce or more complex settings, particularly when guided by variance-based sampling. This difference arises from the presence of an explicit regularization mechanism in vGPC. Whereas exact GPC maximizes the marginal log-likelihood and is therefore driven solely by the data-fit term, vGPC optimizes a variational objective that includes a KL divergence term constraining posterior complexity. This regularization improves generalization in data-scarce regimes, while its influence gradually diminishes as more data are acquired, leading the behavior of vGPC to converge toward that of exact GPC. Unlike the GPC, which relies on a full posterior approximation, the variational formulation of vGPC enables effective regularization through inducing points and approximate inference, resulting in improved generalization under limited data conditions (see Notes S4–S5 for detail). Consistent with these trends, the GPC produced sharper and more confident decision boundaries as more data became available, while vGPC yielded smoother and more stable predictions in the early stages. This behavior is directly reflected in the selection log shown in Figure 2c, which demonstrates that *Autopilot* actively switches between vGPC and GPC depending on data complexity, favoring vGPC under data-scarce or complex conditions and transitioning to GPC as the data set grows and the effective decision surface becomes simpler. For a more detailed view of the selection log, see Supporting Figure S7. Building on the complementary strengths of GPC and vGPC, we designed an adaptive pipeline, *Autopilot*, that dynamically selects the appropriate model based on data characteristics, achieving robust convergence without the need for manual tuning. This adaptive capability, absent in static acquisition approaches, highlights the advantage of integrating dynamic decision-making mechanisms into autonomous experimental platforms.

### Mapping the Synthesizable Space of Au NPs via SPACESHIP and Real Experimental Data: A Binary Classification Approach

Building on the simulation-based insights presented in the previous section, we applied the SPACESHIP framework with *Autopilot* acquisition to a real-world NP synthesis task. The objective was to evaluate the performance of our framework under experimental conditions by conducting a binary classification of synthesizable versus unsynthesizable regions. For this purpose, we implemented the Turkevich approach^35^ for synthesizing Au NPs, in which chloroauric acid (HAuCl_4_) was gradually reduced by sodium citrate at elevated temperatures to yield colloidal Au NPs. This well-established approach offers a controllable and reproducible platform suitable for testing our SPACESHIP framework. In our experimental setting, the synthesizable space was defined by three experimental variables, metal precursor concentration, reductant concentration, and reaction time, as illustrated in Figure 3a. The ground-truth synthesizability landscape was generated from 512 experiments (8 × 8 × 8 grid) and interpolated to a higher-resolution map using a quintic radial basis function. Validation and test sets were held out from this interpolated map prior to training to ensure strict separation between training and evaluation. The labeling function for binary classification is detailed in the Experimental Section. Starting from an initial data set of 20 experiments, SPACESHIP selects subsequent experimental conditions based on model predictions, progressively refining its understanding of the feasible synthesis region.

**Figure 3:**
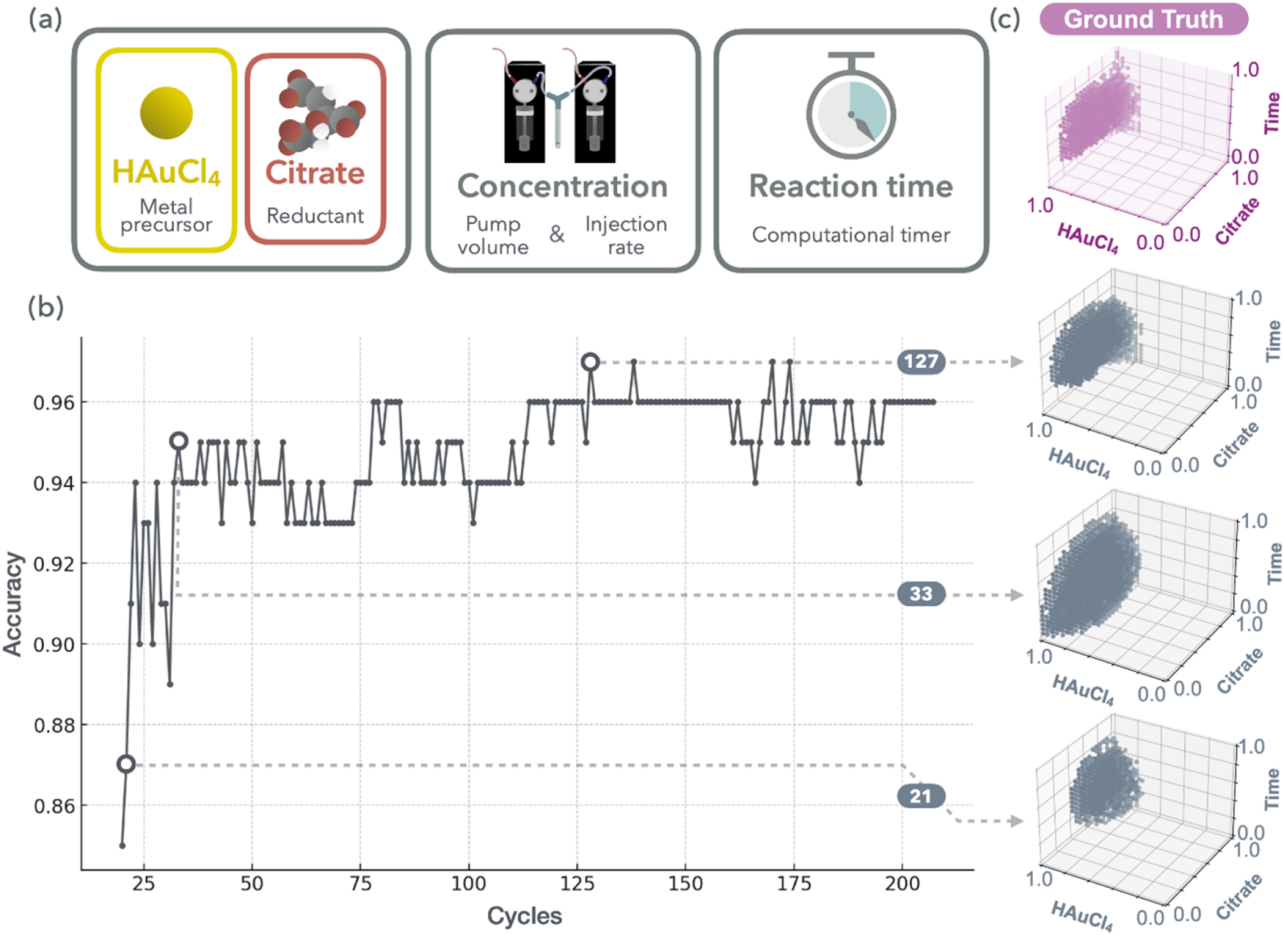
Data-efficient exploration of the synthesizable space of Au NPs using the SPACESHIP framework. (a) Key experimental parameters: precursor type, concentration, and reaction time. (b) Classifier performance as a function of the number of data points, demonstrating rapid convergence. The *x*-axis represents the number of acquisition cycles, and the *y*-axis represents the classification accuracy evaluated on a held-out test set. (c) Progressive refinement of the model’s decision boundary compared with the ground truth, with increasing accuracy of the predicted regions observed as more labeled data are acquired. The *x*- and *y*-axes represent normalized reagent concentrations, and the *z*-axis denotes normalized reaction time. The scatter plots show the predicted decision boundaries generated by the model at each acquisition stage, based on the number of labeled data points indicated in the figure.

Figure 3b shows the improvement in the classification accuracy of SPACESHIP over 200 experimental iterations. The model demonstrated rapid learning, reaching stable performance within the first 130 cycles, indicative of early convergence with limited data (20 sampling data points). In Figure 3c, we compare the evolving decision boundaries predicted by the model to the ground truth boundaries derived from a grid-based set of 512 experiments, performed by our advanced automated hardware,^5^ as described in Figures S8–S9. Over time, the decision boundary of the model increasingly aligned with the true synthesizable region, confirming the ability of SPACESHIP to accurately differentiate synthesizable from unsynthesizable domains. Notably, the decision boundaries did not evolve in a monotonically expanding or contracting manner, but instead adapted dynamically, sometimes expanding, sometimes narrowing, depending on the data acquired at each cycle, underscoring the truly adaptive nature of the framework. These results validate the efficiency of SPACESHIP in generalizing from sparse data to capture complex, nonlinear synthesis constraints. The framework’s data-efficient refinement of synthesizable space highlights its practical utility in accelerating material discovery with minimal experimental effort.

### Application of SPACESHIP to the Morphology-Aware Synthesis of Au NPs: A Ternary Classification Approach

To demonstrate the scalable implementation of SPACESHIP in enabling application-oriented insights into Au NP synthesis, we categorized synthesized Au nanorods (NRs) based on the optical properties, specifically their UV–Vis absorption spectra, into two regimes: visible (λ_max_ ≈ 600–760 nm) and near-infrared (NIR, λ_max_ > 760 nm). This framework aims to solve a ternary classification: visible-range NRs, NIR-range NRs and additional non-NRs, because different localized surface plasmon resonance (LSPR) regions correspond to distinct functional applications.^45,46^ Visible-range Au NRs are typically suited for surface-level imaging, colorimetric sensing, or in vitro biosensing,^47,48^ whereas NIR-range Au NRs are favored in biomedical imaging and photothermal therapy because of their superior tissue penetration and light-to-heat conversion.^49,50^ Using this classification, SPACESHIP enables not only the assessment of NR formation success but also the linkage of synthesis outcomes to specific application domains.

On the basis of this foundation, SPACESHIP mapped the morphology-based synthesizable space by projecting it onto the reagent volumes (Au seed, HCl, HAuCl_4_ and AgNO_3_), revealing distinct regions conducive to NR formation. The labeling function for ternary classification was mentioned in the Experimental Section. According to Figure 4a,b, these mapped regions align with mechanistically grounded trends previously observed in seed-mediated Au NP synthesis. In particular, increasing the Au seed volume expands the NR formation domain (blue and purple regions), likely by increasing the nucleation density and promoting anisotropic growth.^51^ AgNO_3_ influences morphology through facet-selective underpotential deposition of Ag^0^ on {110} facets,^52^ thereby suppressing lateral growth and favoring rod formation over sphere formation. Moreover, HAuCl_4_, as the primary Au precursor, plays a dual role: moderate volumes promote longitudinal elongation, favoring NRs, whereas larger volumes lead to isotropic overgrowth and spherical structures. These SPACESHIP-derived synthesis maps align well with reported observations,^51,52^ thereby validating the model.

**Figure 4:**
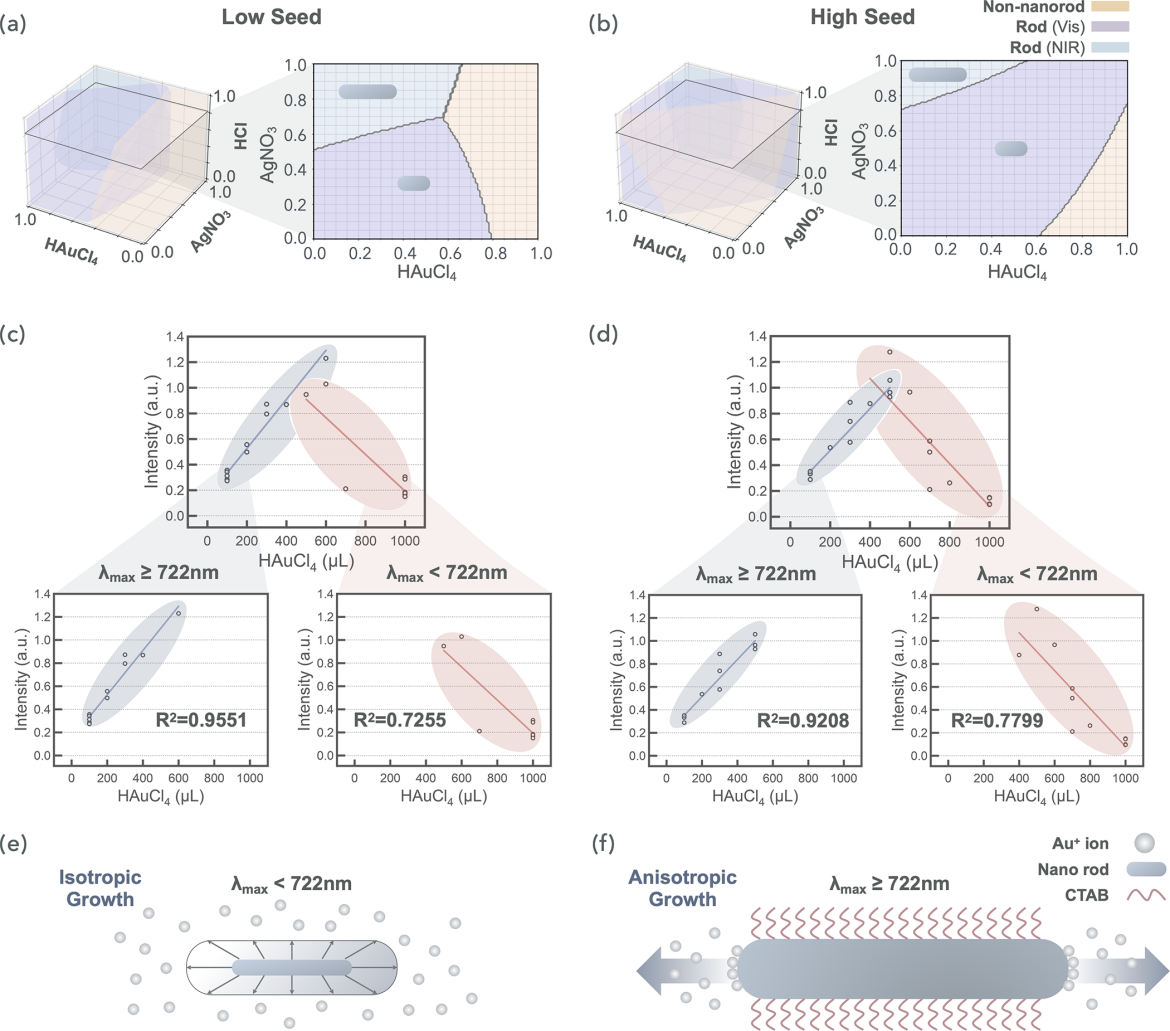
Discovery of chemical insights from the SPACESHIP-guided experimental data. (a, b) Growth diagrams of the Au NRs as a function of the volume of the Au seed solution: (a) 1 mL and (b) 2 mL, with the HCl volume fixed at 0.8 mL. (c, d) Volcano plots illustrating the synthesis outcomes for plasmonic Au NR growth at various Au seed volumes: (c) 1 mL and (d) 2 mL. (e, f) Schematic representations of growth modes at different stages: (e) early stage and (f) late stage.

To further elucidate these morphology trends, we examined the statistical correlations between the reagent volume and optical features. A significant negative correlation was observed between the HAuCl_4_ volume and λ_max_ (Figure S10 and Note S3), indicating that larger precursor volumes reduce the aspect ratios of NRs. This highlights the central role of HAuCl_4_ in modulating NR elongation. Furthermore, the volume of Au seed influenced not only the nucleation density but also the morphological impacts of both HAuCl_4_ and AgNO_3_, increasing the overall sensitivity of NR formation to these reagents.^51^ Partial dependence plots (Supporting Figures S11–S13) further confirmed nonlinear interdependencies among these variables, underscoring the coupled influence of nucleation density, growth directionality, and facet-selective deposition on morphology.

To uncover hidden synthesis-property relationships, we applied causal inference techniques,^53^ which are detailed in the Figure S14. This analysis identified a mechanistic transition boundary at λ_max_ ≈ 722 nm, signifying a shift from isotropic NR growth to anisotropic NR growth. Moreover, a volcano-shaped relationship was observed between HAuCl_4_ volume and plasmonic intensity, with an apex near λ_max_ ≈ 722 nm (Figure 4c,d), delineating the two structural growth stages. In the “early stage” (λ_max_ < 722 nm, Figure 4e), the NRs remain relatively short and have low aspect ratios; increasing the HAuCl_4_ volume at this stage diminishes the plasmonic intensity, likely because enhanced radial growth undermines anisotropy. Conversely, the “late stage” (λ_max_ ≥ 722 nm, Figure 4f) is characterized by anisotropic elongation. Here, increased HAuCl_4_ volumes increase the plasmonic intensity, suggesting that additional precursors promote longitudinal growth at the rod ends, increasing both the aspect ratio and the plasmonic response through enhanced electron confinement along the long axis.^54^ Interestingly, during this late stage, the depletion of Ag on the lateral facets allowed Ag atoms to diffuse into the Au core,^55^ making lateral growth thermodynamically favorable. However, this effect is constrained to the final phase of the reaction, where reduced levels of ascorbic acid and HAuCl_4_ limit growth kinetics. As a result, lateral growth is suppressed, and aspect ratios stabilize through CTAB-mediated surface passivation.^51,56,57^ This dual-stage, volcano-like growth behavior, dependent on NR structural evolution, has not been previously reported. This structural evolution was further confirmed by TEM analysis of synthesized nanorods targeting different LSPR wavelengths (Figure S15), which revealed a progression from isotropic particles to elongated nanorods with increasing aspect ratios and red-shifted plasmonic peaks. Our results, derived via the SPACESHIP framework, not only clarify the mechanism connecting synthesis dynamics and optical properties but also offer a novel paradigm for fine-tuning NR synthesis through control of the Au precursor volume. All the experimental data sets are detailed in Note S3.

### Limitations of Literature-Derived Synthesis Maps and the Advantages of SPACESHIP

In the preceding section, we demonstrated that SPACESHIP enables a comprehensive understanding of the synthesizable spaces of Au NPs and NRs, capturing key features such as growth regimes, phase boundaries, and morphological transitions. However, such insights cannot be derived from the literature alone. This limitation becomes particularly evident when comparing literature-derived synthesis boundaries with those identified using SPACESHIP, as shown in Figure 5. While literature data typically cover only a narrow subset of the experimental design space, SPACESHIP provides a significantly broader and experimentally validated mapping of synthesizable regions. The primary constraint of literature-based data arises from their selective reporting: studies generally focus on successful syntheses, often omitting failed results. This practice leads to an incomplete and biased representation of the synthesizable space. Moreover, these limitations are compounded by system-specific variables, such as differences in reactor design, unreported environmental conditions, or the skill and decisions of individual operators, and by the subjectivity involved in defining “success” according to specific research goals. Conditions deemed unsuccessful in one context may prove valuable for different objectives, particularly when novel or uncharted material systems are explored.

**Figure 5:**
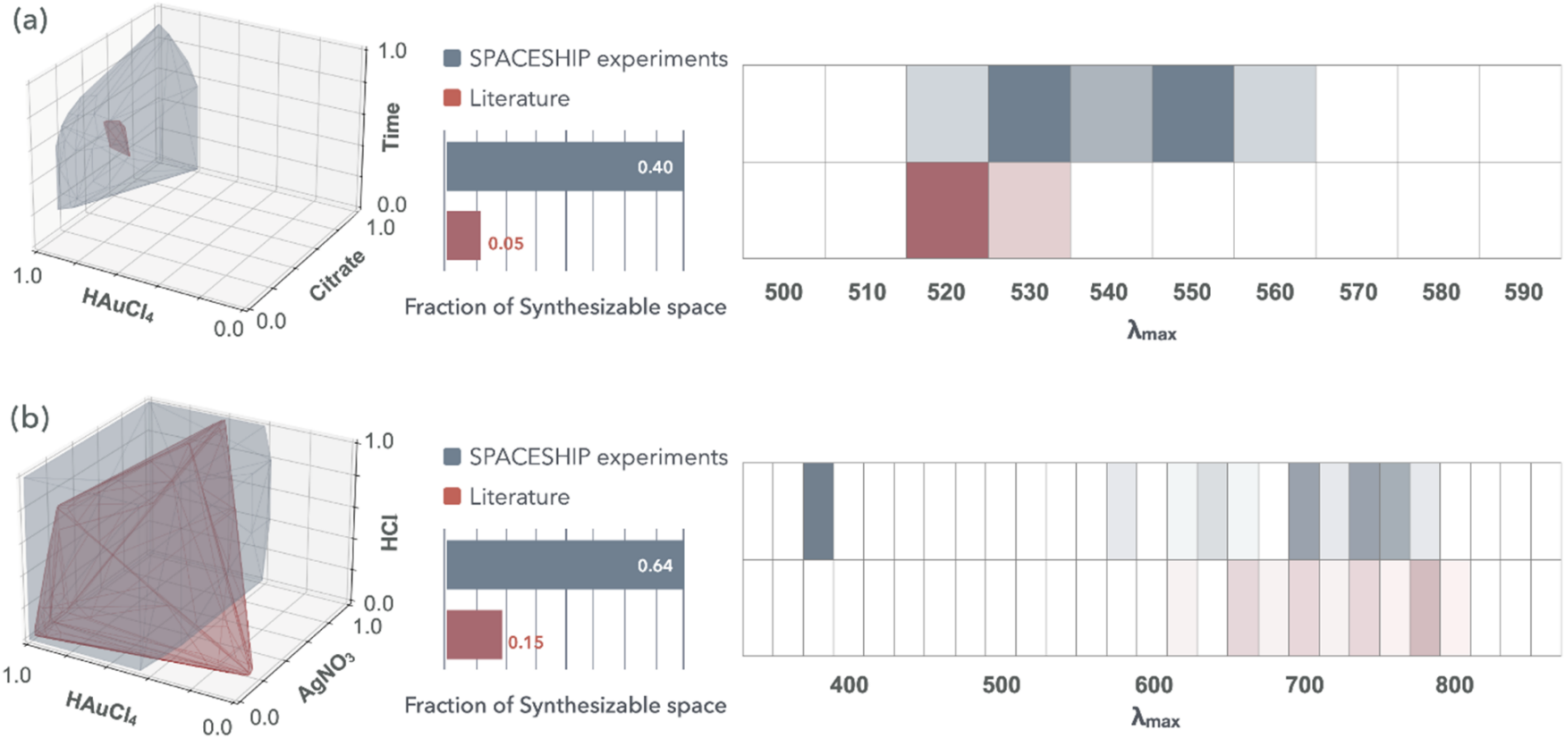
Limitations of literature-based synthesis boundaries. Comparison of synthesizable and property spaces derived from the literature versus SPACESHIP experimental results for (a) Au NP and (b) Au NR synthesis. The isosurface plot represents the normalized synthesizable space. The bar chart represents the fraction of the synthesizable space relative to the total parameter space. The color shade represents the normalized frequency density of the observed properties, with darker regions indicating greater occurrence.

Figure 5a illustrates this disparity: while the literature defines a compact region for Au NP synthesis, SPACESHIP identifies a synthesizable volume nearly eight times larger. A similar trend is observed in Figure 5b, where SPACESHIP expands the synthesizable space for Au NRs by a factor of 4 compared with the literature. These previously uncharted regions may hold critical insights for extending the design boundaries of functional nanomaterials. In addition to differences in synthesizable volume, the UV–Vis spectroscopy data presented in Figure 5a,b reveal that even under nominally identical synthesis conditions, the resulting optical properties can vary significantly. Such variability underscores the influence of hardware-specific factors and undocumented experimental variables, which are rarely captured in published studies. Notably, Figure 5b shows that certain literature-reported conditions for NR formation failed to yield NRs within our experimental hardware. This discrepancy highlights the influence of unreported or hardware-specific variables, beyond reagent identities, that can create misleading constraints when relying solely on prior literature. Such blind spots pose a challenge when designing synthesis protocols for new material targets or transferring methods across platforms. To overcome this limitation, autonomous laboratories need to adopt adaptive frameworks such as the SPACESHIP model, which infer synthesizability constraints directly from experimental feedback rather than relying on fixed, literature-based assumptions.

Further evidence highlights the risks of uncritical reliance on literature-derived data. Variability across studies often results in inconsistent trends and nonreproducible findings, hindering the development of reliable predictive models. As shown in Figure S16, greater variation in synthesis conditions should ideally correspond to predictable changes in experimental results; however, this relationship is far less consistent in literature data sets than in data generated within a single, well-controlled system. Even under identical synthesis conditions, contradictory results are occasionally reported in literature-derived data sets, including those extracted using large language model-based methods,^58^ emphasizing the lack of consistency and reliability.

Additionally, literature-based synthesis maps are often constructed by interpolating between sparsely sampled data points, which can lead to exclusion or feasible synthesis conditions and misclassification of potentially valid regions. In contrast, SPACESHIP integrates both synthesizable and unsynthesizable outcomes and continuously refines its synthesis space using real-time experimental feedback. This enables more accurate, generalizable, and adaptive exploration of the experimental design landscape. By incorporating system-specific constraints and real-time learning, SPACESHIP provides a hardware-aware approach to experimental design. It constructs synthesis maps that are both context sensitive and broadly transferable across different platforms, thereby mitigating the limitations of static, literature-defined boundaries and supporting more robust, exploratory pathways for materials discovery. Nevertheless, the synthesizable space identified by SPACESHIP is not strictly invariant across experimental hardware, reflecting the influence of hardware and system factors on synthesis behavior.

### Adapting Morphology-Aware Synthesizable Spaces to New Experimental Hardware via Transfer Learning

SPACESHIP serves as a general framework for defining a hardware-aware synthesizable space. While the SPACESHIP framework itself is applicable across different experimental hardware configurations, the resulting synthesizable space is not invariant to hardware changes. As shown in Figure 6, comparison of data sets collected using different hot plates reveals that the regions corresponding to feasible morphologies differ even under identical combinations of reaction parameters. These discrepancies arise from hardware-dependent physical factors, such as differences in heating uniformity, heat transfer characteristics, and stirring-induced mixing conditions, all of which directly influence reaction kinetics and nanoparticle growth dynamics.

**Figure 6:**
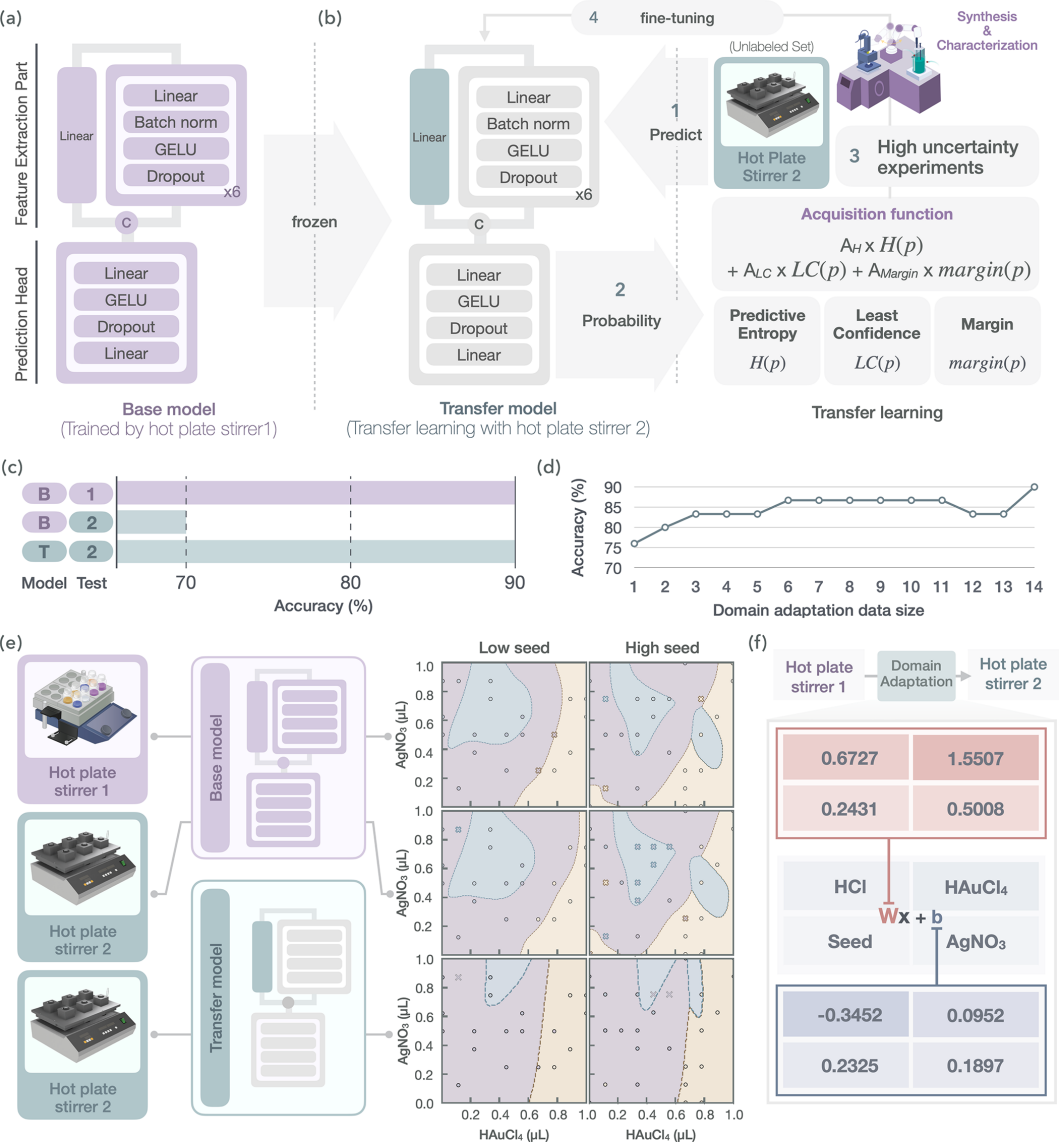
Transfer learning of morphology-aware synthesis models across different hot plate platforms. (a) Architecture of the base model pretrained using data from hot plate stirrer 1. (b) Transfer learning procedure for adapting the pretrained base model to new hot plate stirrer conditions. (c) Comparison of classification accuracy between the base model “B” and the transfer model “T”. The numerical subscripts indicate the hot plate stirrer used for training or evaluation. (d) Classification accuracy as a function of data set size during transfer learning with active learning. (e) Decision boundaries and misclassification distributions for hot plate stirrers 1 and 2. Yellow, purple, and light-blue regions represent non-nanorods, nanorods (Vis), and nanorods (NIR), respectively. Correct and incorrect classifications are denoted by circles “○” and crosses “×”. (f) Hardware-dependent shifts value in synthesizable space for domain adaptation using a linear correction model.

Under such circumstances, reconstructing the synthesizable space from scratch whenever the experimental hardware changes would be highly inefficient in terms of both experimental time and cost. This observation motivates the need for a data-efficient modeling strategy that can adapt morphology-aware synthesis rules across heterogeneous hardware environments while minimizing additional experimentation. The SPACESHIP *Autopilot* introduced in the previous section provides a general framework for morphology-aware space exploration under varying hardware conditions and, a GPC-based surrogate model was employed to efficiently construct the synthesizable space in cold-start scenarios where little to no prior data are available. In this section, we retain the SPACESHIP framework while introducing a modular modeling strategy that allows the surrogate model to be replaced with a transfer learning–enabled alternative when sufficient prior data exist.

Specifically, we adopt a neural network–based modeling framework designed for transfer learning across heterogeneous experimental hardware. In this approach, predictive performance and interpretability are treated as complementary but distinct objectives. The transfer-learned neural network serves as the primary predictive model to rapidly recalibrate the synthesizable space under new hardware conditions, while hardware-induced changes in reaction parameter sensitivities are interpreted using a separate linear correction model. This modular design enhances the reuse of existing experimental data and substantially reduces the experimental effort required to adapt SPACESHIP to new hardware environments.

The model was first pretrained using the morphology-aware training data set obtained during synthesizable space exploration on hot plate stirrer 1, and subsequently adapted to hot plate stirrer 2 through fine-tuning (Figure 6a,b). Here, transfer learning was implemented by freezing the parameters of the gray-shaded layers and fine-tuning only the green-colored linear layers in Figure 6b, allowing the model to adapt to hardware-specific variations while preserving previously learned representations. Fine-tuning was carried out within an active learning framework, in which experimental conditions associated with high predictive uncertainty were preferentially selected. Uncertainty was quantified using a weighted combination of predictive entropy, least confidence, and margin-based criteria, and the detailed formulation of this uncertainty measure is provided in the Experimental Section. The selected experiments were autonomously executed and labeled using an automated batch synthesis module and UV–Vis characterization module, and the newly acquired data were incrementally incorporated into the training set.

Figure 6c summarizes the predictive performance of the transfer learning strategy. When evaluated on hot plate stirrer 1, the base model trained on hot plate stirrer 1 data achieved an accuracy of approximately 90%. However, direct application of the same model to hot plate stirrer 2 without additional training led to a substantial performance drop to approximately 70%, reflecting a pronounced hardware-induced domain shift. In contrast, fine-tuning the model with a limited number of additional experiments from hot plate stirrer 2 restored the prediction accuracy to approximately 90%, demonstrating effective recalibration of the synthesizable space under the new hardware conditions. Notably, this recovery was achieved with only 14 additional active learning cycles, as illustrated in Figure 6d, highlighting the high data efficiency of the proposed approach. Figure 6e visualizes the predicted synthesizable space under both low-seed and high-seed conditions, further confirming that boundaries which differed across experimental hardware were stably reconstructed after fine-tuning, with a marked reduction in misclassified labels.

To interpret the physical origin of the hardware-dependent differences captured by the transfer-learned model, we analyzed a linear correction model as a post hoc interpretability tool rather than as a mechanism for domain adaptation. This correction model was constructed by introducing a linear transformation layer at the input of the existing predictive model and training only this layer using additional data collected under the new hardware condition, while keeping the rest of the model fixed. This design preserves the representations learned by the original model and enables a systematic estimation of hardware-induced shifts in the input reaction conditions. The linear transformation applied to standardized reaction parameters yields feature-wise weights and a bias term that quantitatively describe domain differences between hot plate stirrer 1 and hot plate stirrer 2. In this formulation, features associated with smaller weights require larger adjustments to compensate for hardware-induced effects, while the bias term reflects systematic differences in the absolute input levels required to achieve comparable synthesis outcomes. The extracted parameters, summarized in Figure 6f, provide chemical insight into the origin of hardware-dependent variations in morphology control.

The primary hardware differences between the two systems are likely to stem from differences in their stirring and temperature control mechanisms. Analysis of the correction model reveals that seed concentration, which exhibits the lowest bias term “W” in Figure 6f, is the most influential parameter, indicating that differences in stirring conditions before and after seed injection play a dominant role in shaping the synthesis outcome. A detailed discussion of this mechanism is provided in Note S6. In practice, hot plate stirrer 1 allows the stirring to be turned on and off, whereas hot plate stirrer 2 operates with continuous stirring. Such differences are expected to influence solute transport and local concentration gradients during the initial nucleation stage. This limitation arises from the absence of a communication protocol in hot plate stirrer 2, which prevents external control of stirring and necessitates manual operation via onboard buttons.Additionally, the two systems differ in their temperature control mechanisms. In hot plate stirrer 2, temperature regulation is based on sensor-detected reactor temperature, suggesting that reactions may proceed at a relatively higher effective temperature, leading to increased reaction rates and accelerated growth kinetics. These differences are therefore expected to manifest as systematic variations in particle growth behavior and optical response between the two systems.

Consistent with these observations, synthesis results obtained on hot plate stirrer 1 exhibit an overall red-shift (particle elongation) compared to those from hot plate stirrer 2. This trend is evident in Figure 6e, where comparison of the second and third panels reveals a higher contribution from the long wavelength NIR region (blue-colored area) in samples synthesized on hot plate stirrer 1. Because the linear correction layer is applied upstream of the base model, the input features must be aligned with the conditions under which the base model was trained, namely those of hot plate stirrer 1. In this context, it is important that the model outputs remain consistent with the red-shift trend observed in the experimental data.

To quantify this trend, prediction boundary analysis was performed. When the normalized HAuCl_4_ concentration is fixed at 0.5 and the AgNO_3_ concentration is increased, particle elongation increases up to an optimal concentration and subsequently decreases at higher concentrations, exhibiting nonmonotonic behavior. This suggests that AgNO_3_ promotes facet-selective growth within an appropriate concentration range, whereas excessive AgNO_3_ hinders effective adsorption on specific crystal facets. This interpretation is further supported by the analysis of the bias term (b) in Figure 6f and Note S6. The positive bias associated with AgNO_3_ indicates that a reduced amount of AgNO_3_ on hot plate stirrer 2 is sufficient to achieve shape control comparable to that obtained on hot plate stirrer 1. Moreover, the bias analysis reveals that reproducing the observed red shift on hot plate stirrer 2 requires lower seed concentrations (positive bias) and higher gold precursor amounts (negative bias). This trend is consistent with the well-established growth mechanism^59^ in which fewer initial nuclei allow prolonged particle growth under otherwise identical precursor conditions.

In summary, the proposed transfer learning framework enables efficient adaptation of morphology-aware synthesizable space define models to new hardware system using minimal additional data. Moreover, by jointly employing a transfer learned predictive model and a linear correction model, hardware-induced differences in synthesis behavior can be systematically interpreted from a physical and chemical perspective. This approach provides a practical and generalizable strategy for both transferring synthesizable space define models across experimental systems and understanding the underlying sources of hardware-dependent variability.

## Conclusions

We developed an AI framework, SPACESHIP, and integrated it into an autonomous laboratory for Au NP synthesis. SPACESHIP is designed to explore complex experimental spaces without relying on feasibility constraints derived from literature or expert heuristics, or human supervision. Unlike traditional methods, which begin with a predefined window of “synthesizable” conditions, SPACESHIP dynamically infers synthesizable regions using real-time data, incorporating both successful and failed results to shape and refine its understanding of the search space. This iterative refinement process is driven by a continuously retrained probabilistic model and an uncertainty-aware acquisition strategy that prioritizes exploration of the most informative regions. We demonstrated this capability in the autonomous synthesis of Au NPs, where SPACESHIP accurately distinguished between synthesizable and unsynthesizable regions and effectively targeted specific particle morphologies.

The SPACESHIP framework identifies synthesis regions that are both feasible and aligned with desired objectives without relying on rigid heuristics or manually defined feasibility constraints. By estimating boundaries of feasible regions, it preserves meaningful learning signals and avoids the overrepresentation of uninformative failures, thus reducing the risk of gradient vanishing and enhancing optimization stability. Its design allows users to redefine objectives such as targeting new morphologies, functionalities, or performance metrics by simply updating the labeling function. Furthermore, SPACESHIP supports a hierarchical exploration strategy: low-cost screening experiments eliminate unlikely candidates early, reserving resource-intensive experiments for the most promising conditions. This improves the efficiency of resource allocation and maximizes the scientific value of each experiment.

Beyond optimization, the ability of SPACESHIP to leverage both successful and failed experiments enable deeper scientific inquiry. By systematically analyzing patterns in failed trials, the model can uncover hidden constraints or mechanistic rules not readily deducible from theory, positioning autonomous laboratories as platforms for scientific inference, not just execution. This approach also enables the structured incorporation of literature data. The experimental conditions reported in prior studies, even if not directly aligned with the current system,^60^ can be algorithmically mapped onto the learned reaction space and adapted to the autonomous workflow, preserving their value while respecting system constraints. As new reagents or experimental variables are introduced, the modular architecture of SPACESHIP supports rapid adaptation with minimal additional data, enabling few-shot generalization across broader chemical domains, an aspect we further reflect on in the conclusion.

This work establishes a SPACESHIP framework that elevates autonomous experimentation beyond automation or optimization. It dynamically learns feasible synthesis regions, adapts to evolving experimental conditions, and integrates both success and failure to develop a richer understanding of the underlying chemical landscape. Rather than focusing solely on maximizing material properties, SPACESHIP illuminates unexplored regions of the design space, uncovering new scientific questions that emerge not from what works but rather from what fails. This approach redefines how nanoparticle synthesis is explored, shifting from expert-driven design to truly autonomous, mechanistically guided discovery.

## Experimental Section

### Acquisition Function

For each candidate point *x*, we computed an acquisition score α­(*x*) as a convex combination of the predictive variance from vGPC and the predictive confidence from the GPC

α(x)=λ·vGPC(x)+(1−λ)·GPC(x)

where the weighting parameter λ ∈ [0,1] controls the trade-off between exploration (variance-driven) and exploitation (confidence-driven). The *Cross* strategy uses a fixed weight of λ = 0.5, maintaining equal emphasis on both models. The *Switch* strategy begins with pure variance sampling (λ = 1) and then abruptly transitions to confidence-based sampling (λ = 0) after a predefined cycle threshold is reached. The *Time* strategy implements a gradual transition through a linearly decaying weight, defined as $\lambda =1-\frac{\mathrm{cycle}}{T}$ , where *T* is the total number of exploration cycles. In these three strategies, uncertainty scores from the GPC and variance scores from vGPC are used on the basis of model-specific performance characteristics observed during preliminary evaluations.

In contrast, the *Autopilot* strategy dynamically selects the better-performing model at each acquisition cycle on the basis of validation accuracy and uses its corresponding uncertainty-based score. The decision rule for *Autopilot* is as follows

$$\lambda_{t}=\underset{m\in \left\{\mathrm{GPC},v\mathrm{GPC}\right\}}{\arg\max}{\mathrm{Acc}}_{\mathrm{val}}^{m}\left(t\right)$$

This adaptive mechanism allows *Autopilot* to alternate between exploration and exploitation in response to real-time model performance, optimizing the acquisition process.

For model selection, a separate validation set was used to evaluate the predictive accuracy of each model at every acquisition cycle. In the virtual surface experiments, the validation set consisted of 200 samples, whereas in the real experimental data sets, 50 samples were used for validation. In addition, an independent evaluation set containing 50 samples was reserved exclusively for performance assessment. The evaluation set was never used for model training, validation, or candidate selection during the active learning process. To prevent data leakage in the closed-loop learning setup, the evaluation and validation sets were separated from the initial unlabeled pool at the beginning of the experiment. Specifically, samples assigned to the evaluation and validation sets were popped from the unlabeled data set and appended to dedicated JSON files, where they were stored and managed independently. The remaining samples constituted the candidate pool used for active learning. The training data were maintained separately as a labeled set, ensuring that the evaluation and validation samples were never reintroduced into the training pool during the iterative acquisition process. Sampling of training data was performed using random sampling. To ensure reproducibility, the random seeds used for data set splitting and sampling are specified in the configuration files provided in the GitHub repository.

### Acquisition Function (Transfer Learning)

The acquisition function is defined as a weighted combination of three complementary uncertainty measures derived from the predictive probability distribution of the transfer-learned model

$$A\left(x\right)=\alpha_{H}H\left(x\right)+\alpha_{LC}LC\left(x\right)+\alpha_{Margin}Margin\left(x\right)$$

where, α_H_, α_LC_, and α_Margin_ are tunable coefficients controlling the relative contribution of each uncertainty term. In this study, the coefficients were set to α_H_ = 0.4, α_LC_ = 0.3, and α_Margin_ = 0.3.

The predictive entropy, *H*(*x*), quantifies the overall uncertainty of the model prediction and is defined as

$$H\left(x\right)=-\sum_{i=1}^{K}p_{i}\log p_{i}$$

where *p*_*i*_ denotes the predicted probability of class *i* among *K* possible morphology classes. High entropy values correspond to regions where the model exhibits global uncertainty.

The least confidence term captures the model’s lack of confidence in its most probable prediction

$$\mathrm{LC}\left(x\right)=1-\max_{i}p_{i}$$

This term emphasizes samples for which no single outcome is strongly preferred, highlighting ambiguous regions of the synthesis space.

The margin-based uncertainty focuses on the separation between the two most probable classes

$$\mathrm{Margin}\left(x\right)=1-\left(p_{1}-p_{2}\right)$$

where *p*_1_ and *p*_2_ are the highest and second-highest predicted class probabilities, respectively. Small margins indicate proximity to decision boundaries, which are particularly informative for refining morphology-aware synthesis rules.

By combining these three metrics, the acquisition function balances global uncertainty, local ambiguity, and boundary sensitivity.

### Batch Synthesis Module for Au NPs

#### Materials

Gold­(III) chloride dihydrate (HAuCl_4_·3H_2_O, ≥99.9%), sodium citrate dihydrate (≥99%), sodium borohydride (NaBH_4_, ≥98%), silver nitrate (AgNO_3_, ≥99%), and l-ascorbic acid (≥99%) were purchased from Sigma–Aldrich. Cetyltrimethylammonium bromide (CTAB, ≥99.0%), sodium hydroxide standard solution (NaOH, 1 N), and hydrochloric acid (HCl, 37%) were obtained from Daejung Reagent. The isopropyl alcohol (IPA, 99.9%) used for nanoparticle precipitation was purchased from Samchun. All reagents were used without further purification.

#### One-Pot Au NP Synthesis

For the automated synthesis of Au NPs, we used our autonomous laboratory,^5^ which supports batch-type NP synthesis and UV–Vis characterization. However, for this work, we updated the platform in Figure S7. To support the use of reductants requiring freshly prepared solutions, we integrated a magnetic stirrer (Twister), a capping machine, and an electronic pipet into our system. While the devices themselves were commercially sourced except the capping machine, custom mounting brackets and holders were designed and 3D-printed in-house to secure them in place. These components were manually assembled to fit the specific layout of the platform. For UV–Vis measurements, a detachable gripper was added to enable the use of standard pipet, offering a more flexible alternative to the prior robot–syringe configuration.

During the synthesis of the Au NPs, a robotic arm within our autonomous laboratory system transferred the reagent vials to a designated position on a stirrer machine to ensure consistent and uniform reaction conditions. A total reaction volume of 2,000 mL was prepared by sequentially dispensing the Au precursor (HAuCl_4_·3H_2_O) and sodium citrate dihydrate into the reaction vessel. The concentrations of the reagents and the reaction times were determined by an AI-guided experimental protocol. Reagent delivery was carried out using two micropumps connected through a mixing tee, allowing precise control over the concentration and volume of each solution. The reaction mixture was continuously stirred at 800 rpm and maintained at 80 °C for the AI-recommended reaction time. Following the reaction, the synthesized NP solutions were automatically transferred to the UV–Vis spectroscopy module within our autonomous platform. UV–Vis spectroscopy was then conducted using a built-in spectroscopic analysis system.

### Batch Synthesis Module for Au NRs

#### Materials

Gold­(III) chloride dihydrate (HAuCl_4_·3H_2_O, ≥99.9%), sodium citrate dihydrate (≥99%), sodium borohydride (NaBH_4_, ≥98%), silver nitrate (AgNO_3_, ≥99%), and l-ascorbic acid (≥99%) were purchased from Sigma–Aldrich. Cetyltrimethylammonium bromide (CTAB, ≥99.0%), sodium hydroxide standard solution (NaOH, 1 N), and hydrochloric acid (HCl, 37%) were obtained from Daejung Reagent. The isopropyl alcohol (IPA, 99.9%) used for nanoparticle precipitation was purchased from Samchun. All reagents were used without further purification.

#### Preparation of the Au Seed Solution

A cetyltrimethylammonium bromide (CTAB) solution was first prepared by mixing 9.5 mL of 0.10 M CTAB with deionized water. To this mixture, 0.5 mL of 0.010 M HAuCl_4_·3H_2_O was added under vigorous stirring, and the mixture was stirred continuously for 10 min. Subsequently, 460 μL of freshly prepared, ice-cold NaBH_4_ solution (0.010 M, prepared by dissolving NaBH_4_ in 10 mL of 1 M NaOH) was rapidly injected into the reaction mixture. Upon the addition of NaBH_4_, the color of the solution changed from yellow to yellowish brown, indicating the formation of Au seeds. The mixture was then vigorously stirred for an additional 10 min, after which stirring was halted, and the solution was left undisturbed at 27 °C for 1 h to allow complete seed formation.

#### Seed-Mediated Growth of the Au NRs

For the autonomous synthesis of the Au NRs, the previously prepared Au seed solution, along with stock solutions of the Au precursor (0.010 M HAuCl_4_·3H_2_O), silver nitrate (AgNO_3_, 0.010 M), and l-ascorbic acid (0.010 M), were dispensed into 25 mL vials and placed in designated vial holders on the robotic platform. The Au precursor solution was stored in an opaque vial to prevent photodegradation. The CTAB solutions were prealiquoted into 8 mL portions and stored in a dedicated chemical storage unit. To prevent CTAB solidification, the laboratory environment was maintained at temperatures above 27 °C throughout the experiment. A robotic arm transferred a CTAB vial to a hot plate stirrer maintained at 30 °C where stirring was initiated. For transfer learning experiments conducted on hot plate stirrer 2, a stirrer without on/off control was used, resulting in continuous stirring throughout the synthesis process. Reagents were then added in a sequence determined by an AI-recommended synthesis protocol.

Using a Zeus pipet^61^ mounted on an XYZ actuator,^61^ the robotic system sequentially dispensed the Au precursor and silver nitrate solutions into the stirred CTAB solution. This was followed by the addition of 0.869 mL of prediluted HCl (1 M), which was delivered by the robotic arm^61^ using a twister mechanism. Next, 9.131 mL of ultrapure water was added via a needle connected to a micropump system.^61^ The resulting mixture was stirred for 1 min on the twister platform at 30 °C and then transferred to a reaction vial via a Zeus pipet. l-ascorbic acid solution was subsequently added. After 90 s of additional stirring, the stirrer^61^ was stopped, and the Au seed solution was introduced via a Zeus pipet. The synthesis proceeded at 30 °C for 1.5 h. Upon completion, the vials containing the synthesized Au NRs were automatically transferred to the UV–Vis^62^ spectroscopy module.

#### UV–Vis Characterization Module

After NP or NR synthesis was completed, the robotic arm transferred the reaction vials into a designated vial holder. It then retrieved a cuvette from cuvette storage^61^ and placed it into the UV–Vis spectroscopic module for optical characterization. The cuvette was initially filled with 2 mL of deionized water, followed by the injection of 0.8 mL of the colloidal nanoparticle solution. This was performed using a custom-designed pipet, which was fabricated in-house by modifying a commercially available pipet and mounted onto a custom gripper. To ensure homogeneity, the solution in the cuvette was mixed three times using the same pipetting system. UV–Vis spectral measurements were then conducted via an integrated spectroscopic system. Optical property data, including peak positions and prominences, were extracted using functions from the SciPy library,^63^ particularly *find_peaks* and *peak_prominences*.

### Labeling Function

#### Ground-Truth Synthesizability Map for Au NPs

The discrete experimental outcomes used to construct the ground-truth synthesizability landscape were obtained from an 8 × 8 × 8 grid of reaction conditions (512 experiments in total) spanning normalized precursor concentration, reductant concentration, and reaction time. These experimentally measured points formed the sole basis for constructing the ground-truth model.

To obtain a continuous approximation of the synthesizability space, the three-dimensional grid coordinates were reshaped into an (N, 3) array and paired with the corresponding binary synthesis outcomes. A smooth three-dimensional interpolant was trained using the RBFInterpolator (SciPy) with a quintic radial basis function and a smoothing parameter of 0.1. This interpolant was then evaluated on a denser 21 × 21 × 21 grid spanning the same normalized coordinate ranges, yielding 9,261 uniformly spaced points, which were thresholded at 0.5 to produce a binary synthesizability map. At the start of the study, the parameter space was partitioned into disjoint training and test pools. During active learning, models were trained only on conditions selected from the training pool, while all evaluation metrics were computed exclusively on the held-out test pool. Neither the original 512 experimental points nor the interpolated values were used to seed or pretrain the models. Interpolation was introduced to enable systematic benchmarking and visualization of decision boundaries, as exhaustive experimental sampling of the continuous parameter space would require an astronomically large number of physical experiments.

#### Labeling for Binary Classification of Au NPs

The raw UV–Vis spectra were cropped to the 300–849 nm range, corresponding to the emission range of the deuterium and halogen light sources used in the system. To reduce noise, a boxcar smoothing filter with a box size of 10 was applied to the cropped spectra. Peak detection was performed using a prominence threshold of 0.01 and a minimum full width at half-maximum (FWHM) of 20 nm. Spectra containing at least one peak satisfying these criteria were labeled successful syntheses, whereas those without qualifying peaks were labeled unsuccessful.

#### Labeling for the Ternary Classification of Au NRs

The maximum absorption wavelength, λ_max_, was extracted from the UV–Vis spectrum to classify the synthesized Au NPs according to their optical characteristics. Au NRs are characterized by two distinct absorption peaks: one located below 550 nm (transverse mode) and another above 550 nm (longitudinal mode).^62^ The presence of a peak at or above 550 nm served as the primary criterion for identifying nanorod samples. If a spectral peak was observed at a wavelength of λ_*i*_ = 550 nm, the sample was classified as a nanorod, and λ_max_ was defined as the wavelength of that peak. Conversely, if no peak was detected in this region, the sample was considered a non-nanorod (label = 0), and λ_max_ was considered undefined. This classification rule is summarized as follows

$$\lambda_{\max}=\{\begin{array}{l} {\begin{array}{l} \lambda \end{array}}_{i},\mathrm{if}\,\;{\begin{array}{l} \lambda \end{array}}_{i}\geq 550\;\mathrm{nm}\;\mathrm{and}\;a\;\mathrm{peak}\;\mathrm{exists}\\ \mathrm{None},\mathrm{if}\,\;\mathrm{no}\,\;\mathrm{peak}\;\mathrm{in}\;\begin{array}{l} \lambda \end{array}\geq 550\;\mathrm{nm} \end{array}$$

The nanorod-classified samples were further categorized on the basis of the value of λ_max_. If λ_max_ fell within the range of 550–760 nm, the sample was labeled a visible-absorbing NR (label = 1). If λ_max_ exceeded 760 nm, it was labeled a near-infrared (NIR)-absorbing NR (label = 2). The full classification scheme is as follows

$$\lambda_{\max}=\{\begin{array}{l} \lambda_{i}\left(visible\right),{if\;550\leq \lambda }_{i}\leq 760\;nm\\ \lambda_{i}\left(NIR\right),if\;\lambda_{i}>760\;nm \end{array}$$

This classification procedure forms a labeling function that assigns samples into one of three distinct categories—non-NR, NR-visible, and NR-NIR—on the basis of their UV–Vis absorption spectra. This systematic categorization supports its subsequent data analysis and enables multiclass active learning for autonomous experimentation.

### Causal Effects of Synthesis Parameters on Optical Properties

The causal effects^64^ of the synthesis parameters on the optical properties of the Au NPs were systematically estimated across various spectral conditions. We analyzed UV–Vis peak data obtained from train data of active learning, which include peak wavelength and peak intensity measurements together with experimental conditions (HAuCl_4_, AgNO_3_, HCl, and Seed). All variables were standardized using z-score normalization prior to analysis to ensure comparability of effect sizes across predictors and outcomes. To investigate potential structural changes in treatment effects across the spectral domain, we conducted a systematic sweep over candidate wavelength cutpoints. For each cutpoint c, the data were partitioned into a low-wavelength region (Zone 1, λ ≤ *c*) and a high-wavelength region (Zone 2, λ > *c*). Cutpoints were evaluated at 1 nm increments within the observed wavelength range, excluding 20 nm from each boundary to reduce edge effects, and only cutpoints yielding at least eight observations per zone were retained.

For each treatment–outcome pair and cutpoint, standardized linear regression models were fit separately within each zone to estimate zone-specific treatment effects while adjusting for the remaining experimental variables as covariates. Candidate “effect flips” were defined as cutpoints at which the estimated treatment coefficients had opposite signs between Zone 1 and Zone 2. To account for the large number of tested cutpoints, a joint *p*-value was defined conservatively as the maximum of the two zone-specific *p*-values, and false discovery rate (FDR) control was applied using the Benjamini–Hochberg procedure with a target level of *q* = 0.05.

For causal effect estimation, treatments were dichotomized within each zone using the median of the standardized treatment variable, thereby contrasting relatively high versus low treatment levels. Covariate balance between treated and control groups was assessed using standardized mean differences (SMDs) for all nontreatment covariates, and both the median and maximum SMD values were recorded for each zone. Propensity scores were estimated via logistic regression using the remaining experimental variables as predictors. Overlap and positivity were further evaluated by inspecting the propensity score distributions and by quantifying the proportion of observations with extreme propensity scores (<0.05 or >0.95).

The primary causal estimate was the average treatment effect (ATE), estimated using stabilized inverse probability weighting (IPW). Stabilized weights were constructed from the estimated propensity scores, and weighted least-squares regression of the outcome on treatment indicator was performed with heteroskedasticity-consistent (HC3) standard errors. To assess sensitivity to limited overlap, analyses were repeated after trimming observations with propensity scores outside the interval [0.1, 0.9]. In addition, robustness was evaluated using alternative estimators, including 1:1 nearest-neighbor matching on the propensity score scale and overlap weighting, which down-weights observations with extreme propensity scores without explicit trimming.

To evaluate robustness to potential violations of the ignorability assumption due to unmeasured confounding, *E*-values were computed for each zone-specific standardized treatment effect. *E*-values quantify the minimum strength of association, on the risk ratio scale, that an unmeasured confounder would need to have with both treatment and outcome to fully explain away the observed effect. Finally, consistency of the estimated treatment effect direction was examined across multiple estimation strategies to ensure that identified effect reversals were not artifacts of a single modeling approach.

### TEM Analysis

Following NP synthesis, 0.5 mL of the sample was transferred to a 1 mL microcentrifuge tube and diluted with an equal volume (0.5 mL) of deionized water. The diluted solution was sonicated at 13,500 rpm for 10 min. After sonication, the supernatant was carefully removed to avoid disturbing the precipitate. The remaining pellet was redispersed in deionized water to a final volume of 1 mL, and this washing step was repeated twice to remove residual impurities. The purified NPs were then redispersed in 1 mL of IPA for TEM analysis. A carbon-coated copper grid (200 mesh) was placed on filter paper inside a Petri dish, and 5 μL of the prepared sample was drop-cast onto the grid two or three times to ensure adequate particle deposition. Morphological characterization and length measurements of the Au NRs were performed via transmission electron microscopy (TEM) (FEI Technai F20 G20).

## Supplementary Material



## Data Availability

Code is available at https://github.com/KIST-CSRC/SPACESHIP.

## Notes

Benchmark validation and model selection details, automated hardware design, partial dependence and causal analyses of synthesis parameters, TEM and optical characterization of Au nanorods, experimental data sets with literature comparison, and descriptions of machine learning models including the linear correction model for domain adaptation (PDF)
