# Supplementary material for: SPACESHIP: Autonomous Mapping of Hardware-Dependent Synthesizable Space in Solution-Phase Gold Nanomaterials
Source: J Am Chem Soc. 2026 Apr 29;148(18):19170–85. doi: 10.1021/jacs.6c03132 (PMC13184979; doi:10.1021/jacs.6c03132)
Supplement: Supplementary file 1 [file ja6c03132_si_001.pdf]

Supporting Information

SPACESHIP: Autonomous Mapping of Hardware-dependent Synthesizable Space in Solution-Phase Gold Nanomaterials

Nayeon Kim,[1,2,†] Hyuk Jun Yoo,[1,†] Daeho Kim,[1,3] Heeseung Lee,[1,4] Chang Seop Hong,[2] and Sang Soo Han[1,]*

[1]Computational Science Research Center, Korea Institute of Science and Technology, Seoul 02792, Republic of Korea

[2]Department of Chemistry, Korea University, Seoul 02841, Republic of Korea

[3]Department of Chemical and Biological Engineering, Korea University, Seoul 02841, Republic of Korea

[4]Department of Materials Science and Engineering, Korea University, Seoul 02841, Republic of Korea

Keywords: Autonomous lab, Explore Synthesizable space, Active Learning, Mechanistic Discovery via AI, Beyond Literature-Derived Constraints

Supporting Figures

Figure S1. Construction of the parameter space from experimental conditions and hardware specifications.......... 4
Figure S2. Visualization of Olympus benchmark surfaces and their transformation into binary classification spaces by thresholding.......... 5
Figure S3. Performance comparison between the GPC and vGPC with increasing data complexity.......... 7
Figure S4. Performance comparison between the GPC and vGPC on the HyperEllipsoid. 8
Figure S5. Performance comparison between the GPC and vGPC on the Rosenbrock. 9
Figure S6. Performance comparison between the GPC and vGPC on the Dejong....... 10
Figure S7. Selection log of Autopliot between GPC and vGPC. 11
Figure S8. Advanced hardware components of the automated batch synthesis module. 12
Figure S9. CAD structures of the experimental hardware components.......... 13
Figure S10. Correlation between $HAuCl_4$ and λ_{max} as a function of volume of Au seed: (left) 1,000 μL and (right) 2,000 μL 14
Figure S11. Partial dependence plots for non-nanorods. 15
Figure S12. Partial dependence plots for nanorods in visible region. 16
Figure S13. Partial dependence plots for nanorods in NIR region. 17
Figure S14. Robust causal estimation of Au NR properties across propensity-score regions.......... 18
Figure S15. TEM images of gold nanorods synthesized at targeted LSPR peak wavelengths using the SPACESHIP model. Scale bar: 20 nm.......... 19
Figure S16. Binned analysis of optical output variation as a function of condition distances.......... 20

Supporting Tables

Table S 1. Threshold settings for classification of Olympus benchmark functions. 22
Table S 2. Percentage of parameter space explored to identify the synthesizable region using random sampling with various machine learning models.......... 23
Table S 3. Correlation metrics of λ_{max} as a function of $HAuCl_4$ volumes. 24

Table S 4. Experimental dataset of synthesis parameters and optical measurements for Au nanorods. Each entry is labeled according to the type of nanoparticles synthesized: Non-nanorod=0, Vis-Nanorod=1, and NIR-Nanorod=2.................. 25
Table S 5. Reported synthesis conditions for citrate-stabilized Au nanoparticles from literatures.. 27

Supporting Notes

Supporting Figures

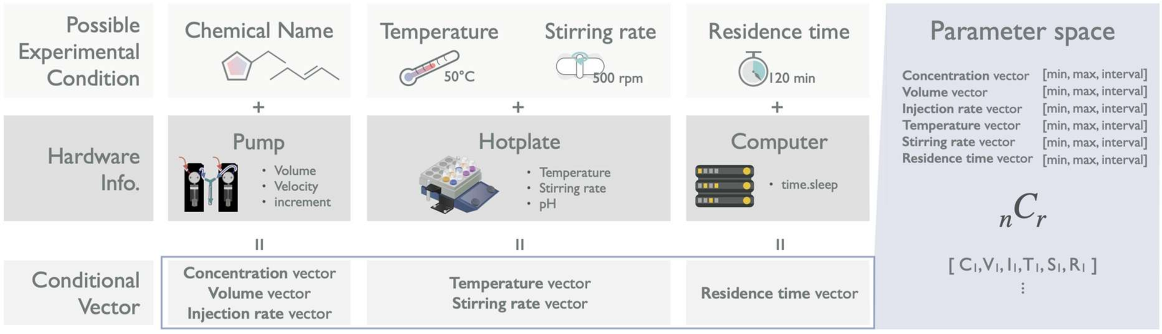

Figure S1. Construction of the parameter space from experimental conditions and hardware specifications.

To define the parameter space in a manner that is both interpretable by AI models and executable by laboratory hardware, we constructed it on the basis of a combination of experimental conditions and hardware specifications. Since experimental conditions are inherently dependent on the hardware configuration, they were encoded into condition vectors. For example, data such as chemical identity and pump configuration were translated into three types of condition vectors: concentration, volume, and injection rate. When systematically combined, these vectors define the complete parameter space. This structured representation enables efficient and comprehensive exploration of experimental conditions within an autonomous laboratory framework.

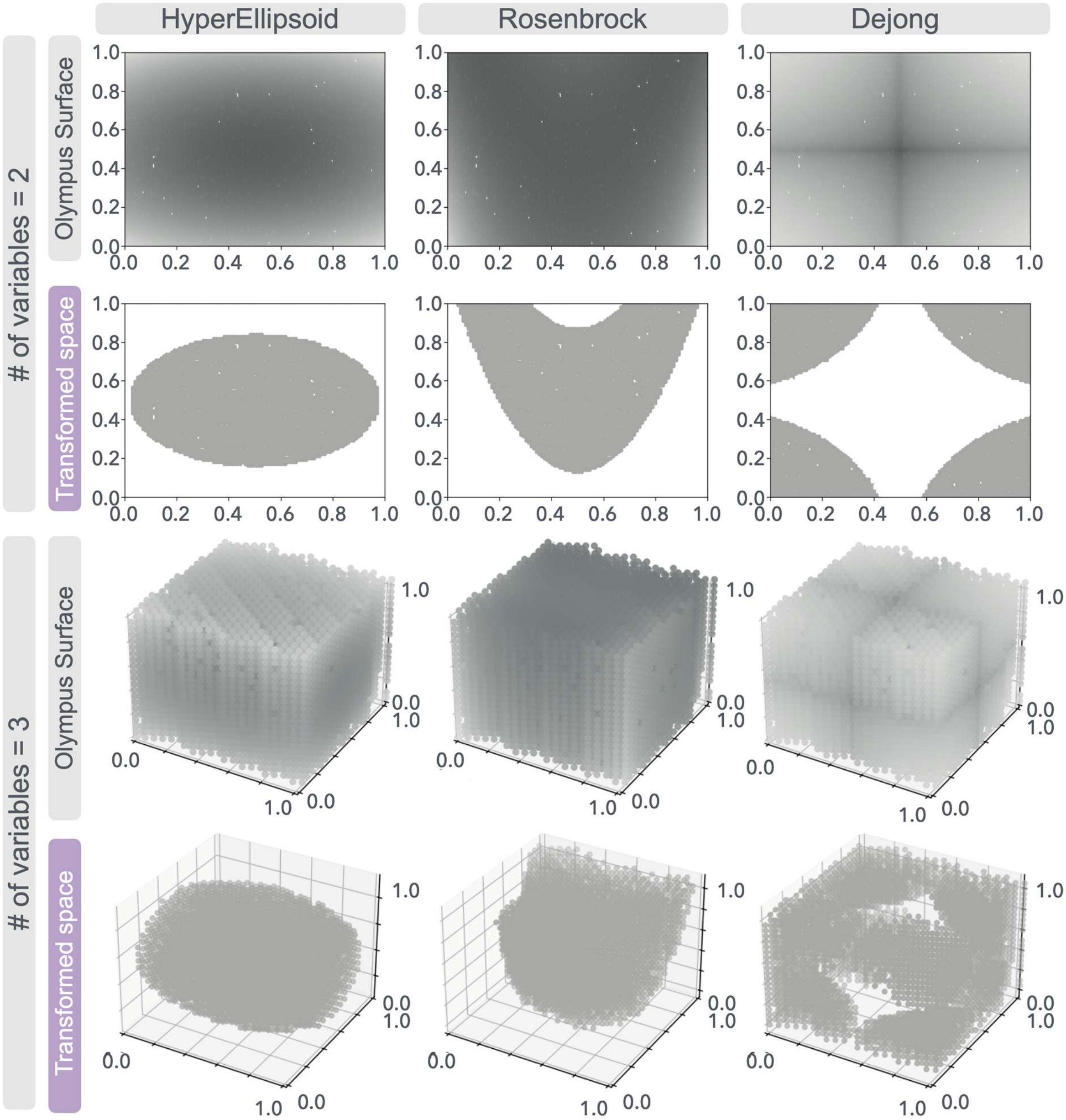

Figure S2. Visualization of Olympus benchmark surfaces and their transformation into binary classification spaces by thresholding.

This figure compares the original continuous-valued Olympus benchmark surfaces (top and third rows) with their corresponding binary classification maps obtained through thresholding (second and bottom rows). Each column represents a different benchmark function—HyperEllipsoid, Rosenbrock, and Dejong—while each pair of rows shows results for 2-variable (top half) and 3-

variable (bottom half) configurations. A fixed threshold was applied to segment each surface into synthesizable (gray) and unsynthesizable (white) regions, effectively converting the original continuous-valued surfaces into binary classification spaces. This transformation enables constraint-aware model evaluation by reframing the task as binary classification rather than regression.

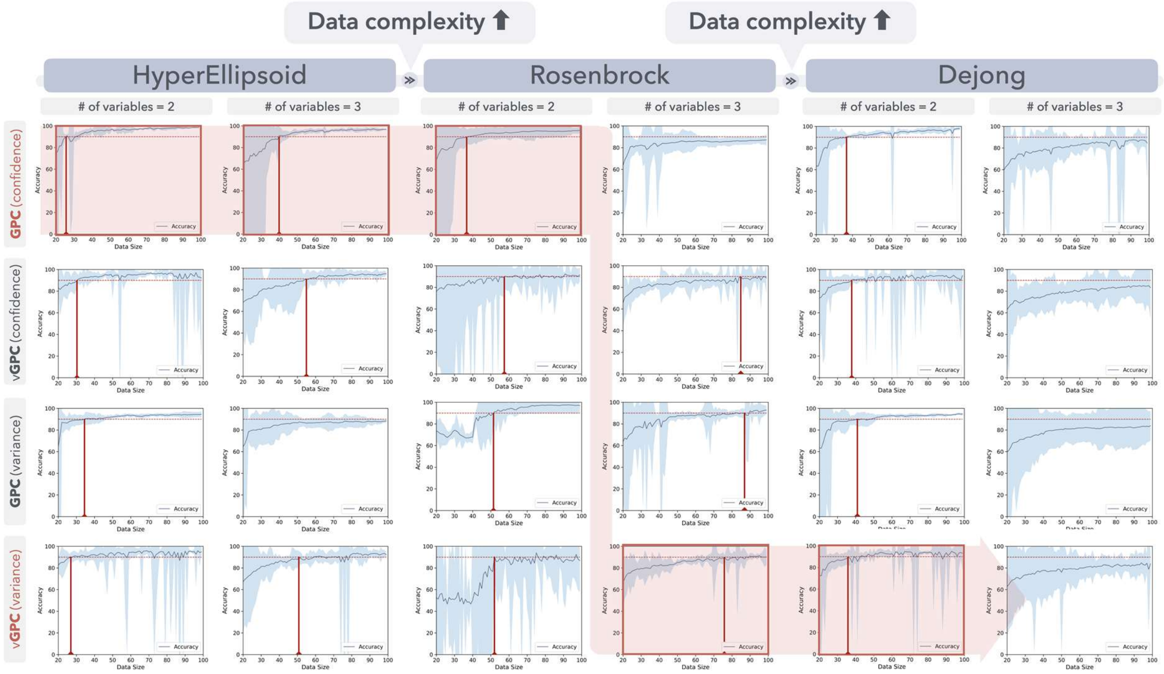

Figure S3. Performance comparison between the GPC and vGPC with increasing data complexity.

This figure presents classification accuracy trends of the GPC and vGPC on three Olympus benchmark functions of increasing complexity—HyperEllipsoid, Rosenbrock, and Dejong—across two input dimensions (2D and 3D). Each subplot illustrates accuracy as a function of the number of labeled data points, using either confidence-based or variance-based acquisition strategies. The results indicate that the GPC is more effective for simpler functions or when ample labeled data are available, particularly with confidence-based acquisition. Conversely, vGPC excels in more complex or data-scarce settings, with variance-based acquisition consistently outperforming confidence-based approaches. The model that reached 95% performance the fastest is highlighted with a red box in the figure. This analysis highlights the importance of tailoring model choice and acquisition strategies to data complexity and availability, providing practical guidance for adaptive model selection in active learning workflows. For detailed plots, see Supporting Figures 4–6.

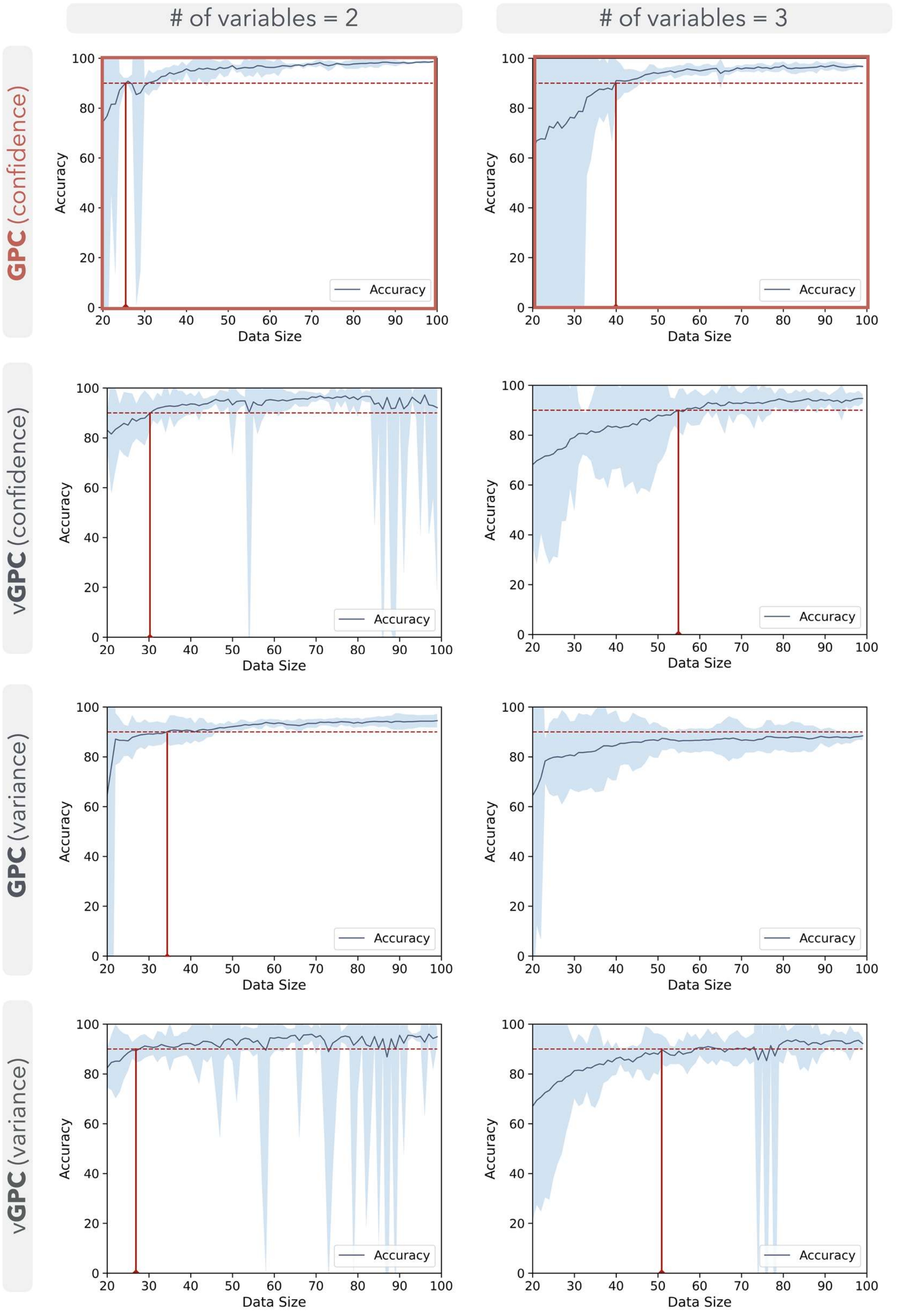

Figure S4. Performance comparison between the GPC and vGPC on the HyperEllipsoid.
The model that reached 95% performance the fastest is highlighted with a red box in the figure.

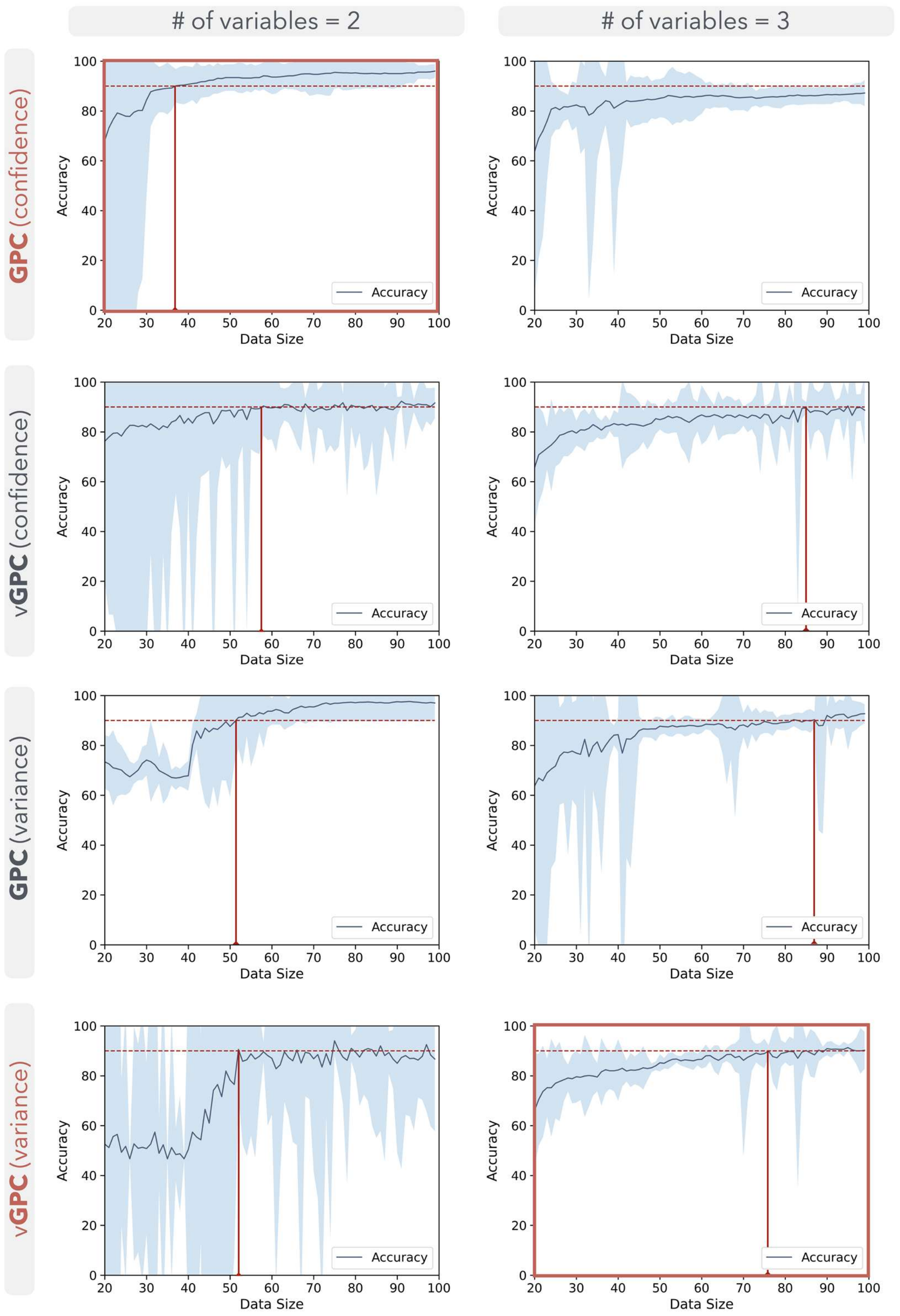

Figure S5. Performance comparison between the GPC and vGPC on the Rosenbrock.

The model that reached 95% performance the fastest is highlighted with a red box in the figure.

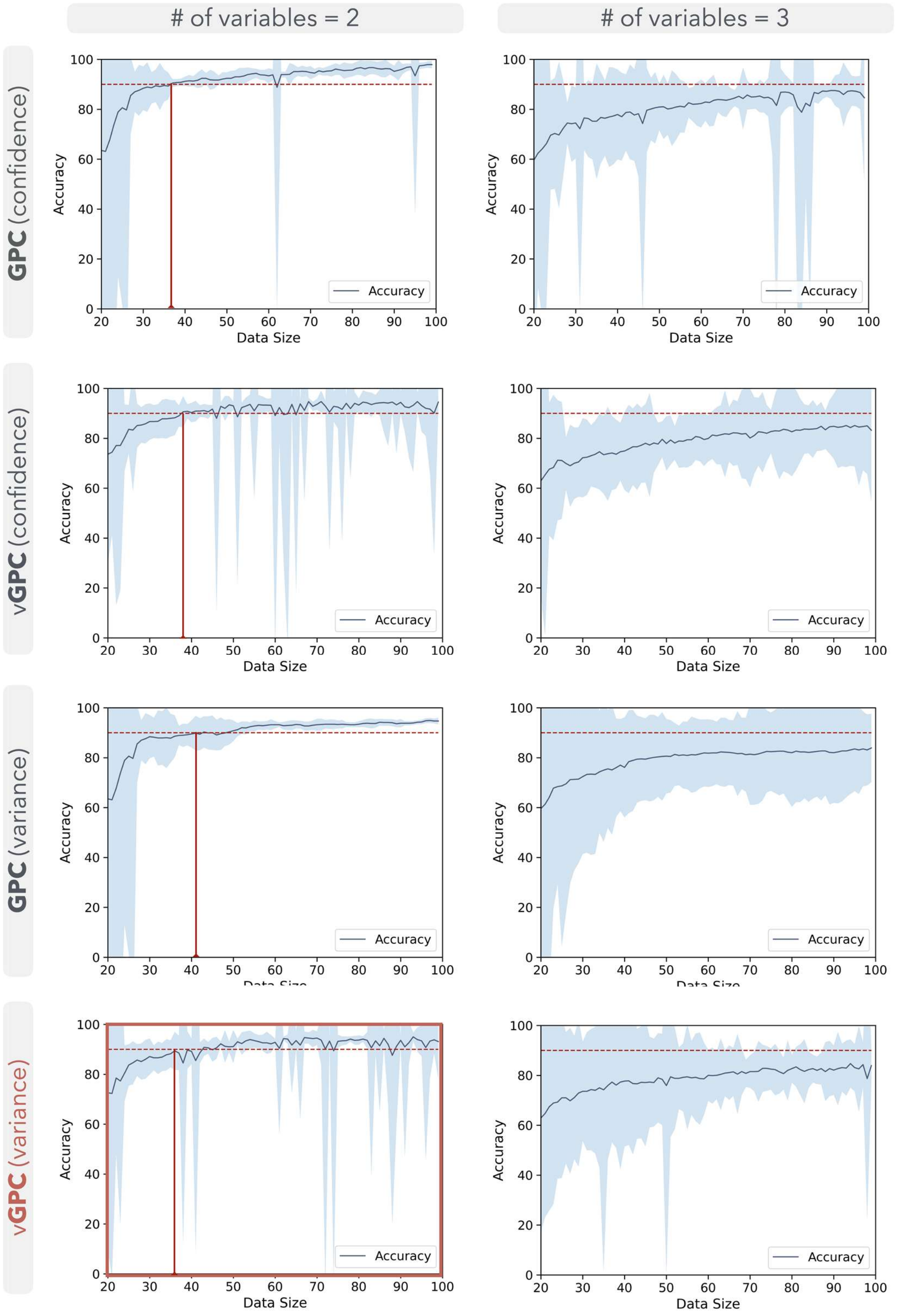

Figure S6. Performance comparison between the GPC and vGPC on the Dejong.

The model that reached 95% performance the fastest is highlighted with a red box in the figure.

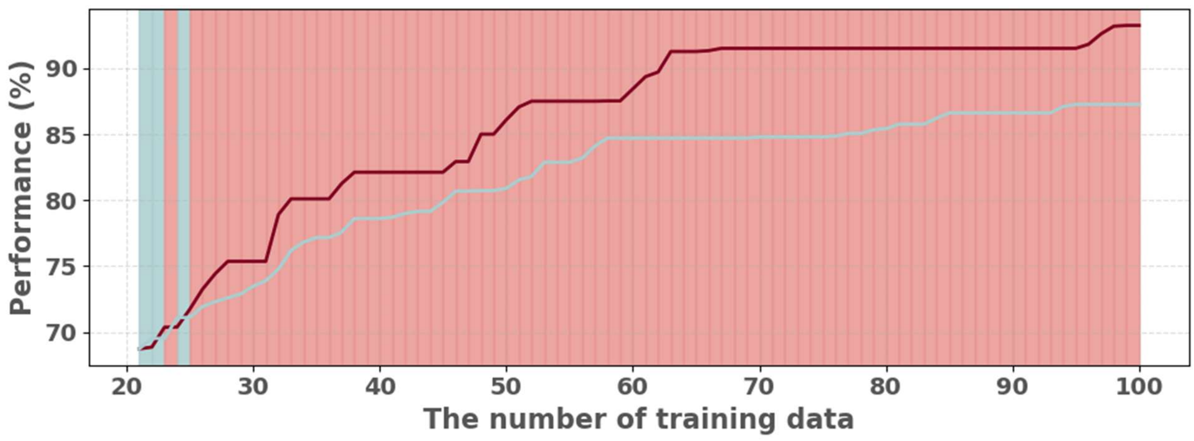

Figure S7. Selection log of Autopliot between GPC and vGPC.

This figure visualizes the cycle-wise switching behavior of the Autopilot mechanism, where the background color indicates which model is selected at each training step (red for GPC and blue for vGPC). The overlaid performance curve shows the model accuracy as a function of the number of acquired training samples. The plot illustrates that early in training Autopilot alternates between GPC and vGPC as uncertainty estimates differ substantially, whereas after sufficient data accumulation the system stabilizes and consistently selects the more reliable expert.

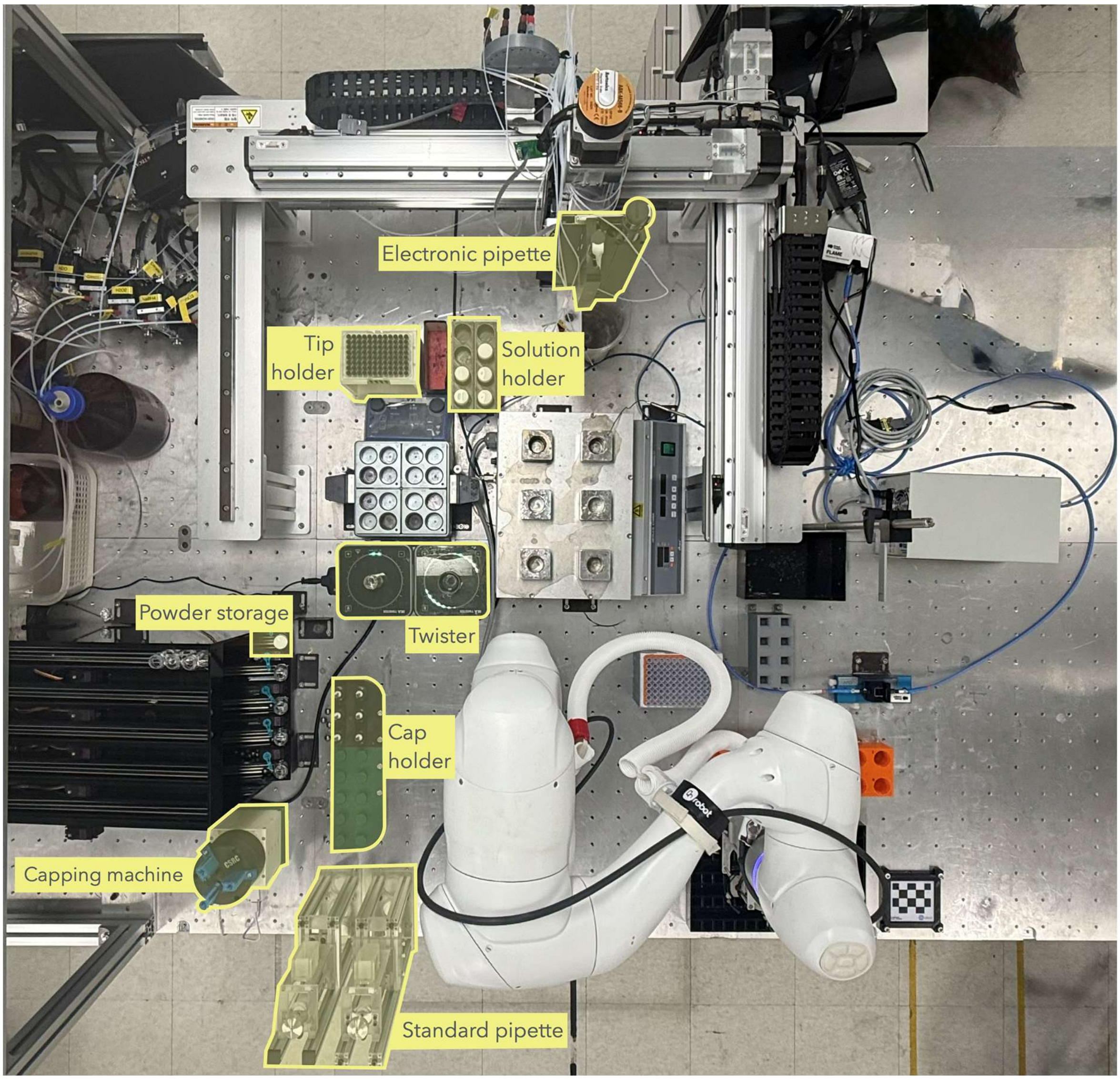

Figure S8. Advanced hardware components of the automated batch synthesis module. Labeled box of the automated synthesis module showing newly integrated hardware devices highlighted in yellow. Recent advances include a twister, cap holder, capping machine and electronic pipette to enable solution preparation from powder-type reductants. In addition, a gripper-mounted standard pipette was incorporated to expand the module's liquid handling capabilities, allowing for optional operation. These modifications were implemented to support reproducible solution handling under fully automated control.

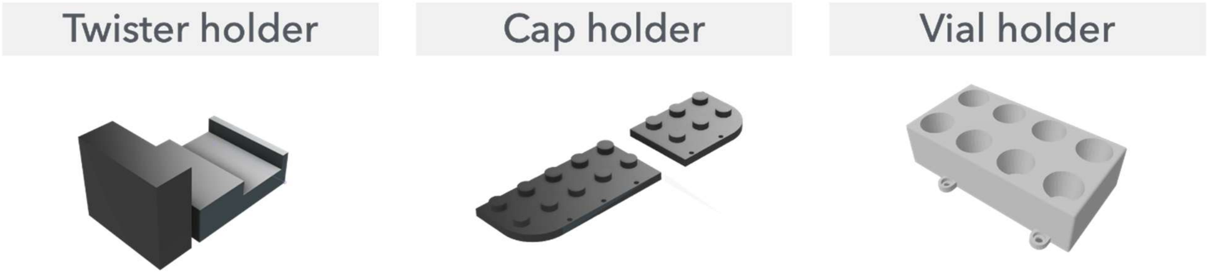

Figure S9. CAD structures of the experimental hardware components.
(Left) Twister holder, (middle) cap holder for capping machine handling highly reactive solutions, and (right) vial holder.

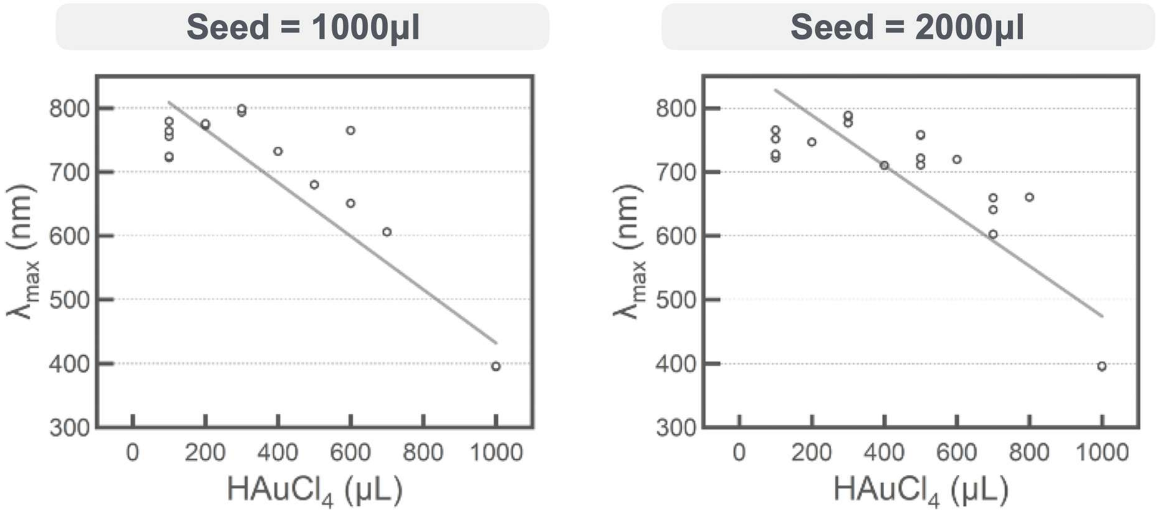

Figure S10. Correlation between $HAuCl_4$ and λ_{max} as a function of volume of Au seed: (left) 1,000 µL and (right) 2,000 µL

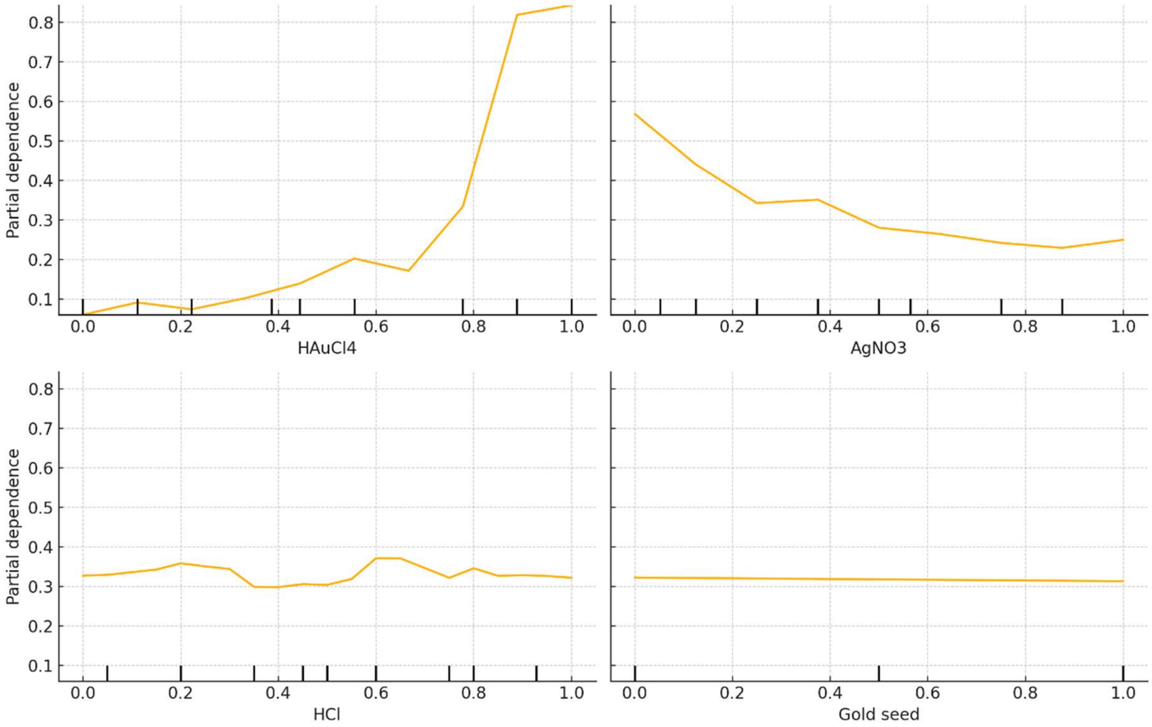

Figure S11. Partial dependence plots for non-nanorods.

This figure shows the partial dependence plots of each chemical variable ($HAuCl_4$, $AgNO_3$, HCl, and Au seeds) with respect to the probability of forming a non-nanorod product. The x-axis represents the normalized volume of each reagent, and the y-axis indicates the model-predicted probability of non-nanorod formation. This result reveals that large normalized $HAuCl_4$ volumes strongly promote non-nanorod outcomes, whereas increasing amounts of $AgNO_3$ and Au seeds reduce the likelihood of such outcomes. In contrast, the HCl volume has a negligible influence. These trends suggest that isotropic growth is favored when $HAuCl_4$ dominates in the absence of sufficient shape-directing reagents.

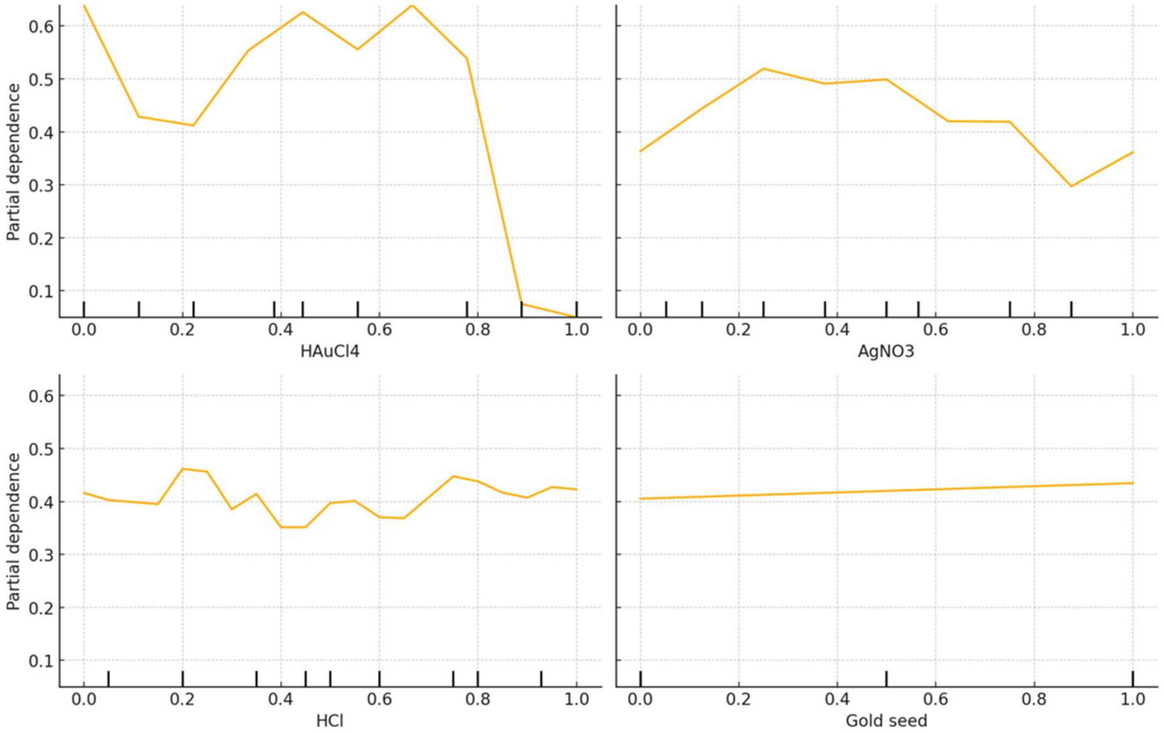

Figure S12. Partial dependence plots for nanorods in visible region.

This figure shows the partial dependence plots of each chemical variable with respect to the probability of forming a nanorod in the visible region. The x-axis represents the normalized volume of each reagent, and the y-axis indicates the model-predicted probability for nanorod(vis) formation. The results indicate that large normalized volumes of $HAuCl_4$ reduce the probability of forming nanorods, whereas increased levels of $AgNO_3$ and Au seeds increase this probability. The HCl volume had minimal influence. These trends suggest that anisotropic growth is not favored when $HAuCl_4$ dominates in the absence of sufficient shape-directing agents.

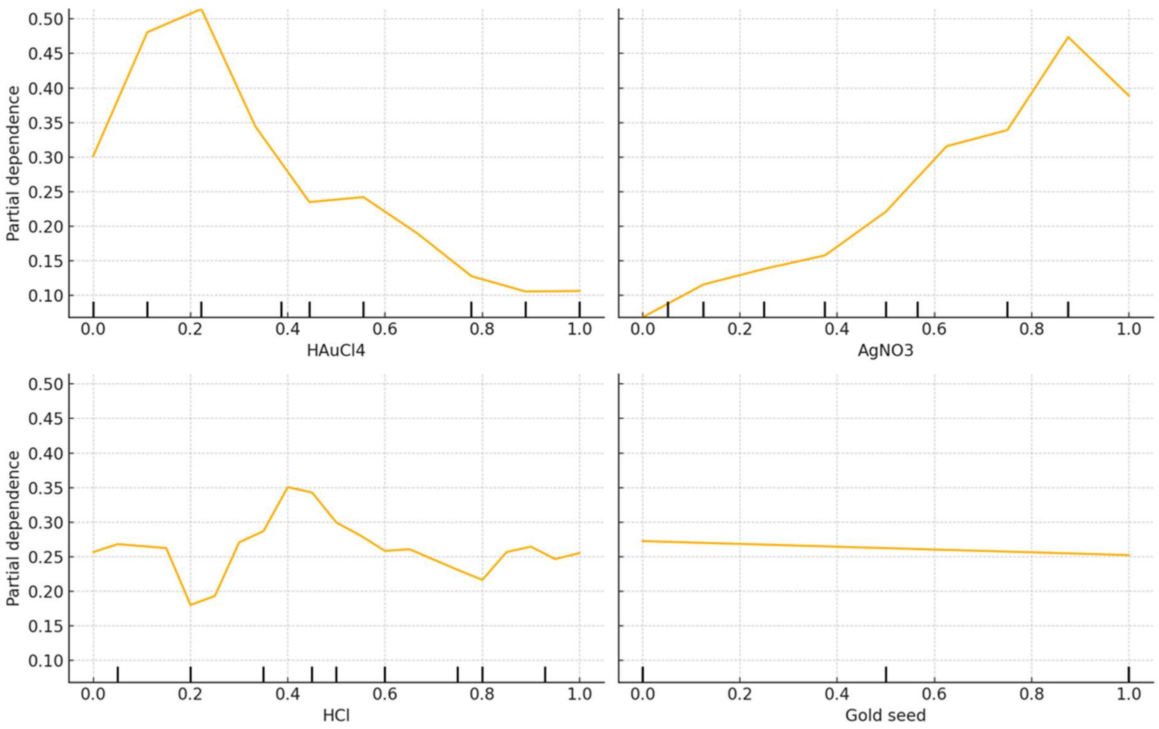

Figure S13. Partial dependence plots for nanorods in NIR region.

This figure illustrates the partial dependence plots for nanorod formation in the NIR region. The x-axis represents the normalized volume of each reagent, and the y-axis indicates the predicted probability of forming a nanorod with NIR optical features. A combination of small $HAuCl_4$ volumes and large volumes of $AgNO_3$ and Au seeds significantly increased the likelihood of nanorod formation. This trend is consistent with the anisotropic growth promoted by silver ion-mediated facet control and the abundance of nucleation sites provided by the Au seeds.

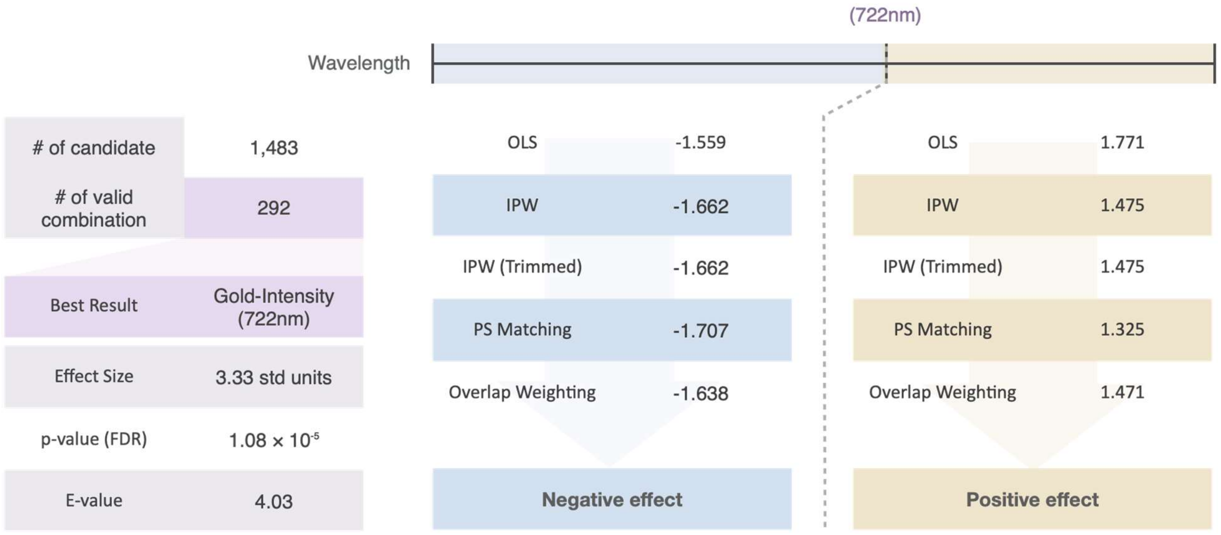

Figure S14. Robust causal estimation of Au NR properties across propensity-score regions. Screening across wavelengths identified 722 nm as the strongest treatment–outcome signal among 1,483 candidates and 292 valid combinations. The standardized effect size for Gold–Intensity is 3.33 SD units with strong statistical significance after FDR correction (adjusted p = 1.08×10^{-5}) and an E-value of 4.03, indicating robustness to potential unmeasured confounding. Zone-specific causal effects were estimated to account for differences in propensity-score overlap at all estimators (OLS, IPW, trimmed IPW, PS matching, and overlap weighting) consistently showed negative effects in Zone 1 and positive effects in Zone 2. The convergence of effect directions across these five independent methods demonstrates strong ignorability and overlap supportive evidence and confirms that the identified causal effect is stable across heterogeneous regions of the propensity score distribution.

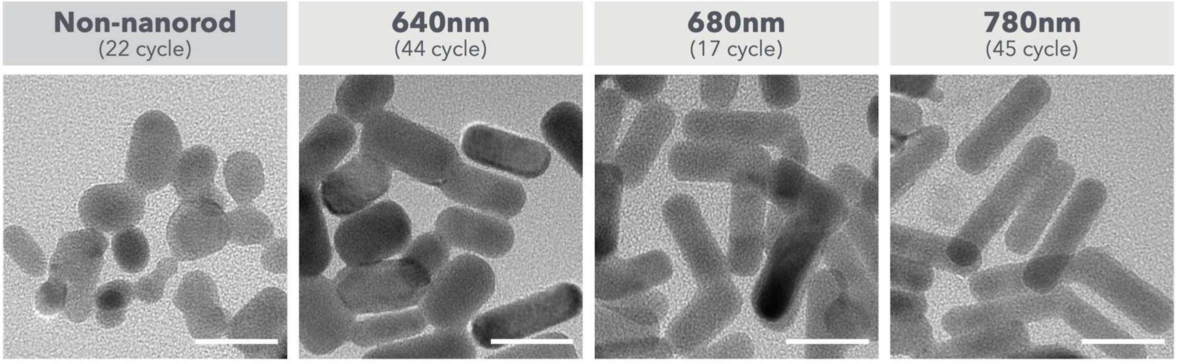

Figure S15. TEM images of gold nanorods synthesized at targeted LSPR peak wavelengths using the SPACESHIP model. Scale bar: 20 nm.

Transmission electron microscopy (TEM) images showing the morphological evolution of gold nanoparticles synthesized under the guidance of our SPACESHIP model, with longitudinal surface plasmon resonance (LSPR) wavelengths of 640 nm, 680 nm, and 780 nm. The number of experimental iterations required to achieve each target is indicated in parentheses. The early iteration (leftmost panel) displays polydisperse, isotropic particles prior to anisotropic growth. In the UV–Vis spectrum, non-nanorod samples are identified by the lack of a characteristic double peak above 550 nm. The corresponding TEM images often show a mixture of spherical particles and early-stage morphologies that have not undergone full anisotropic growth into nanorods. With successive synthesis cycles, nanorods with progressively higher aspect ratios are formed, corresponding to longer LSPR wavelengths. The results highlight the system's ability to autonomously navigate the design space toward anisotropic nanostructures with tunable optical properties.

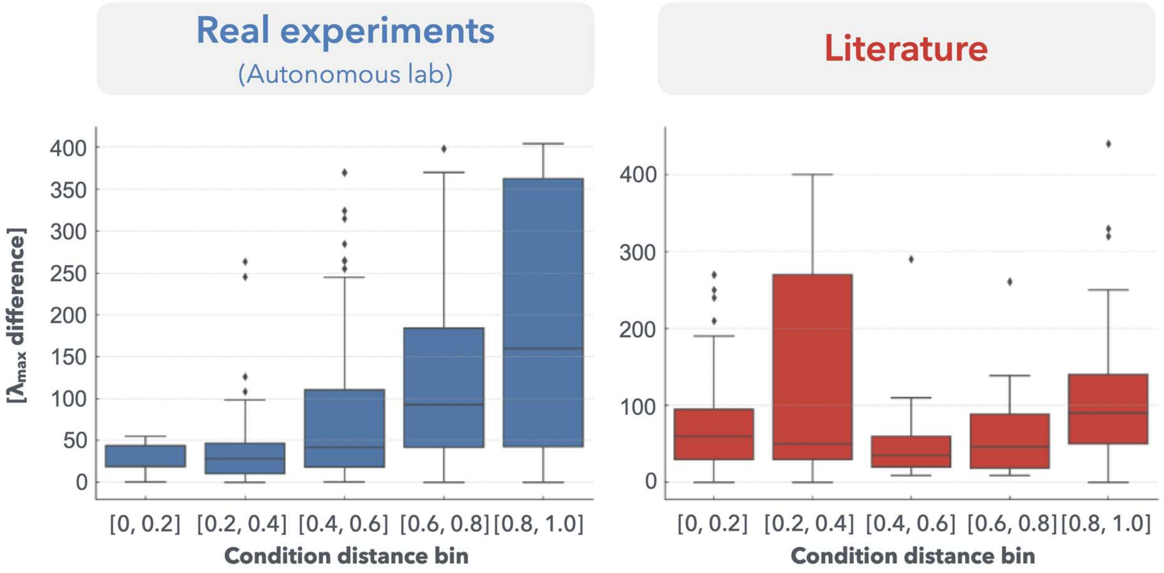

Figure S16. Binned analysis of optical output variation as a function of condition distances. This figure shows how variation in synthesis conditions correlates with changes in optical properties considering the hypothesis that larger differences in input conditions should lead to greater differences in output. The condition distance was calculated as the Euclidean distance between condition vectors, whereas the property distance was measured as the absolute difference in λ_{max}. The x-axis represents the normalized condition distance, binned at intervals of 0.2, and the y-axis indicates the median and interquartile range (IQR) of λ_{max} differences within each bin. The results from the SPACESHIP experiments reveal a statistically significant positive correlation between condition distance and λ_{max} variation, suggesting reliable sensitivity of outputs to experimental inputs. In contrast, the literature data exhibit no correlation trend, likely due to interlaboratory variability and unreported experimental factors. These findings highlight the limitations of relying solely on literature-reported conditions for autonomous synthesis, as they may not reliably reproduce the reported optical properties.

Supporting Algorithm

Algorithm 1: Autopilot — Active Learning with Validation-Based Model Selection

Data: Parameter space $\mathcal{X}$, full unlabeled pool $\mathcal{D}_{pool}$, objective function $f(x)$, initial sample size n_0, budget b

Result: Updated labeled dataset $\mathcal{D}_L$ after b evaluations

`/* Initialization: separate held-out sets before learning begins */`

Pop validation set $\mathcal{D}_{val}$ from $\mathcal{D}_{pool}$;

Pop evaluation set $\mathcal{D}_{eval}$ from $\mathcal{D}_{pool}$;

Assign remaining samples as unlabeled candidate set $\mathcal{D}_U \leftarrow \mathcal{D}_{pool}$;

Randomly sample n_0 points from $\mathcal{D}_U$, evaluate $f(x)$, and initialize $\mathcal{D}_L$;

`/* Active learning loop */`

while *evaluation budget not exhausted* **do**

- Train two surrogate models on $\mathcal{D}_L$: a Gaussian process classifier $\mathcal{M}_{GPC}$ and a variational Gaussian process classifier $\mathcal{M}_{vGPC}$;
- Evaluate both models on $\mathcal{D}_{val}$ and select the best-performing model $\mathcal{M}^*$ based on validation accuracy;
- **foreach** $x \in \mathcal{D}_U$ **do**
  - **if** $\mathcal{M}^* = \mathcal{M}_{vGPC}$ **then**
    - $\alpha(x) \leftarrow \max_k P(y{=}k \mid x; \mathcal{M}^*)$;
  - **else**
    - $\alpha(x) \leftarrow 1 - \left[P(\hat{y}_1 \mid x; \mathcal{M}^*) - P(\hat{y}_2 \mid x; \mathcal{M}^*)\right]$;
- Select $x_{next} = \arg\max_{x \in \mathcal{D}_U} \alpha(x)$;
- Evaluate objective $y_{next} = f(x_{next})$;
- Update $\mathcal{D}_L \leftarrow \mathcal{D}_L \cup (x_{next}, y_{next})$;
- Remove x_{next} from $\mathcal{D}_U$;
- Update budget $b \leftarrow b - 1$;

Algorithm S1. Active learning with validation-based model selection and feasibility-aware acquisition.

The algorithm describes a closed-loop active learning procedure that adaptively selects the most suitable classifiers (e.g., GPC and vGPC) on the basis of validation performance. At each iteration, candidate points from the unlabeled pool are scored using an acquisition function derived from the current best-performing model. A feasibility constraint (e.g., synthesizability) is then applied to filter out infeasible candidates prior to evaluating the objective function. This approach ensures efficient use of the experimental budget by prioritizing both informative and practically achievable samples.

Supporting Tables

Table S1. Threshold settings for classification of Olympus benchmark functions.

	Hyperellipsoid		*Rosenbrock*		*Dejong*	
	# of Variable = 2	*# of Variable = 3*	*# of Variable = 2*	*# of Variable = 3*	*# of Variable = 2*	*# of Variable = 3*
Min/Max	0.0077/75.0	0.0765/167.8145	0.0076/3609.0	0.3452/7218.0	0.4495/4.4721	0.8387/6.8856
Threshold	23	52	220	700	3.1	4.6

This table indicates the minimum and maximum values of each Olympus benchmark surface (Hyperellipsoid, Rosenbrock, and Dejong) in both 2-variable and 3-variable configurations. To enable classification-based model training and evaluation, each continuous-valued function was converted into a binary classification task using a predefined threshold. Data points with objective values below the threshold were labeled as "synthesizable," while those above were labeled "unsynthesizable." Thresholds were chosen to balance class distribution and ensure representative coverage of the objective space.

Table S2. Percentage of parameter space explored to identify the synthesizable region using random sampling with various machine learning models.

	Logistic regression[1]	*MLP*	*XGBoost*	*GP Ensemble*	*TabPFN*	*vGPC*	*GPC*
Fraction of the explored space (%)	-	6.182 ±4.678	0.317 ±0.148	0.324 ±0.295	0.159 ±0.151	0.095 ± 0.078	0.091 ±0.084

This table reports the fraction (%) of the total parameter space that was explored to successfully identify the synthesizable region for the Olympus benchmark problems. The evaluated models include Logistic Regression, Multi-Layer Perceptron (MLP), XGBoost, Gaussian Process (GP) Ensemble, TabPFN, variational Gaussian Process Classifier (vGPC), and Gaussian Process Classifier (GPC). A dash (–) indicates cases where the explored fraction exceeded 10%, reflecting poor selectivity or unreliable model performance. All values are reported as mean ± standard deviation over multiple independent trials. The results compare different machine learning models under random sampling strategies, highlighting their efficiency in discovering feasible regions within the complete search space.

Table S3. Correlation metrics of λ_{max} as a function of $HAuCl_4$ volumes.

Au Seed (μL)	***R-Square***	***Pearson's R***
1,000	0.8641	-0.9295
2,000	0.7463	-0.8639

Table S4. Experimental dataset of synthesis parameters and optical measurements for Au nanorods. Each entry is labeled according to the type of nanoparticles synthesized: Non-nanorod=0, Vis-Nanorod=1, and NIR-Nanorod=2.

idx	*$HAuCl_4$ (μL)*	*$AgNO_3$ (μL)*	*HCl (μL)*	*Au Seed (μL)*	*λ_{max} (nm)*	*FWHM (nm)*	*Label*
1	400	100	300	2000	746.8354	0.535823	1
2	800	30	260	2000	719.6475	0.966241	1
3	500	30	300	1000	793.2933	0.795533	2
4	900	60	180	1000	775.7247	0.556056	2
5	1000	70	250	2000	727.8174	0.332546	1
6	600	70	130	2000	395.016	0.149358	0
7	600	30	250	1000	755.8024	0.273173	1
8	300	90	200	2000	751.7886	0.333659	1
9	300	20	170	2000	395.3781	0.178527	0
10	1000	20	190	1000	764.1075	0.342743	2
11	900	50	100	1000	395.016	0.184045	0
12	200	30	180	2000	765.6409	0.288749	2
13	600	30	140	1000	602.4149	0.500681	1
14	100	40	110	2000	724.3655	0.285136	1
15	900	20	220	1000	395.3781	0.145383	0
16	500	20	160	2000	395.7401	0.149811	0
17	900	30	100	1000	680.1679	0.94744	1
18	200	20	260	2000	757.3435	0.928911	1
19	100	90	110	1000	732.5134	0.868561	1
20	500	20	100	1000	774.5058	0.49786	2
21	400	100	300	2000	395.3781	0.168135	0
22	800	30	260	2000	788.7687	0.576596	2
23	500	30	300	1000	396.1021	0.094527	0
24	900	60	180	1000	772.9809	0.49744	2
25	1000	70	250	2000	660.4391	0.262262	1

26	600	70	130	2000	395.7401	0.097244	0
27	600	30	250	1000	395.3781	0.098395	0
28	300	90	200	2000	722.1653	0.350827	1
29	300	20	170	2000	659.4637	0.586326	1
30	1000	20	190	1000	765.0277	1.230024	2
31	900	50	100	1000	799.0054	0.872588	2
32	200	30	180	2000	710.807	1.277019	1
33	600	30	140	1000	650.6634	1.029269	1
34	100	40	110	2000	758.2676	0.962139	1
35	900	20	220	1000	721.8508	0.96352	1
36	500	20	160	2000	606.0884	0.210929	1
37	900	30	100	1000	710.1738	0.876839	1
38	200	20	260	2000	395.7401	0.286927	0
39	100	90	110	1000	758.5754	1.057366	1
40	500	20	100	1000	722.4798	0.356303	1
41	400	100	300	2000	776.6383	0.886659	2
42	800	30	260	2000	786.3501	0.738901	2
43	500	30	300	1000	395.3781	0.304546	0
44	900	60	180	1000	640.84	0.211304	1
45	1000	70	250	2000	779.3758	0.312323	2

Table S5. Reported synthesis conditions for citrate-stabilized Au nanoparticles from literatures.

Literatures	*HAuCl4 (mM)*	*Citrate (mM)*	*Reaction time (min)*	*Wavelength (nm)*
Turkevich et al., 1951 [4]	1.0	4	30	520
Frens, 1973 [5]	0.25	0.25	30	530
	0.25	0.5	30	525
	0.25	0.75	30	520
	0.25	1.0	30	518
	0.25	1.25	30	517
Haiss et al., 2007 [6]	0.296	0.513	40	530
	0.296	0.556	40	526
	0.296	0.599	40	522
	0.296	0.642	40	519
	0.296	0.684	40	517
Panariello, 2020 [7]	0.2	2.4	5.5	520
Chavva et al., 2024 [8]	0.125	0.0102	22	533.7
	0.125	0.0136	22	531.3
	0.125	0.0170	22	527.0
	0.125	0.0255	22	521.0
	0.125	0.0289	22	519.0
	0.125	0.0340	22	519.0
Sánchez et al., 2006 [9]	1.5	3	20	520
Sun & Xia, 2002 [10]	1.0	4	30	520
Kimling et al., 2006 [11]	0.25	1.0	15	520

This table summarizes synthesis conditions of gold nanoparticle (Au NP) from selected literature sources employing citrate-based reduction methods, similar to those used in this study. Each entry includes the concentrations of gold precursor ($HAuCl_4$) and citrate, reaction time, and the resulting surface plasmon resonance peak wavelength (λ_{max}). These data provide a comparative basis for evaluating the reproducibility, spectral characteristics, and kinetics of Au NP synthesis, and serve

as a reference for assessing the consistency and generalizability of our autonomous synthesis platform navigated by the SPACESHIP model relative to established methods.

Supporting Notes

Note S1. Logistic regression

Logistic regression[1] models the probability of class membership via a linear function of the input:

$$P(y = 1 \mid \mathbf{x}) = \sigma(\mathbf{W}^{\mathrm{T}}\mathbf{x} + b) \quad where \quad \sigma(z) = \frac{1}{1 + e^{-z}}$$

Here, $\mathbf{x} \in R_{dx}$ denotes the input vector (reagent volumes), $\mathbf{w}$ is the weight vector, and b is the bias term. The model parameters are estimated by minimizing the regularized binary cross-entropy loss:

$$\mathrm{L} = -\frac{1}{N}\sum_{i=1}^{N}[y_i log\hat{y}_i + (1 - y_i)\log(1 - \hat{y}_i)] + \lambda\|\mathrm{w}\|^2$$

where λ is the L2 regularization strength. We used **L-BFGS**[12] optimization and set $\lambda = 1.0$. This model provides interpretability via the learned weights, representing the influence of each input variable.

Note S2. Multilayer Perceptron

The Multilayer Perceptron(MLP)[2] is a feed-forward neural network consisting of multiple layers of linear transformations followed by non-linear activations. A two-layer MLP can be represented as:

$$\hat{\mathrm{y}} = \mathrm{softmax}(\mathbf{W_2} \cdot \mathrm{ReLU}(\mathbf{W_1}\mathbf{x} + \mathbf{b_1}) + \mathbf{b_2})$$

where $\mathbf{W}_1, \mathbf{W}_2$ are weight matrices, and ReLU is the rectified linear unit $\mathrm{ReLU(z)} = \max(0, \mathrm{z})$. The model is trained to minimize the cross-entropy loss between the predicted softmax output and true class labels:

$$\mathcal{L} = -\sum_{i=1}^{N}\sum_{c=1}^{C} y_{ic} log\hat{y}_{ic}$$

Dropout regularization with rate 0.2 was applied after each hidden layer. Optimization was performed using Adam with a learning rate of 0.001 and a batch size of 32.

Note S3. XGBoost

XGBoost[3] is a gradient-boosted decision tree algorithm that builds an ensemble of additive regression trees. At each boosting round t, a new tree f_t is added to minimize the regularized objective:

$$\mathcal{L}^{(t)} = \sum_{i=1}^{N} l\left(y_i, \hat{y}^{(t-1)} + f_t(\mathbf{x}_i)\right) + \Omega(f_t) \quad where \quad \Omega(f) = \gamma \mathrm{T} + \frac{1}{2}\lambda \sum_{j=1}^{T} \omega_j^2$$

Here, l is typically the softmax cross-entropy loss, $\Omega(f)$ penalizes model complexity via number of leaves T and leaf weights ω_j. The algorithm uses second-order Taylor expansion to compute gradient and Hessian statistics at each node.

We used 100 boosting rounds, tree depth of 3, and a learning rate of 0.1. Early stopping was triggered after 10 rounds without validation improvement.

Note S4. Gaussian Process Classifier (GPC)

The Gaussian Process Classifier (GPC) models the latent function $f(x)$ as a Gaussian process prior:

$$f(x) \sim GP\big(0, k(\mathbf{x}, \mathbf{x}')\big)$$

where k is the Matérn 5/2 kernel with Automatic Relevance Determination (ARD), defined as:

$$k(\mathbf{x}, \mathbf{x}') = \sigma^2 \left(1 + \frac{\sqrt{5}r}{\ell} + \frac{5r^2}{3\ell^2}\right) \exp\left(-\frac{\sqrt{5}r}{\ell}\right)$$

$$r = \|\mathbf{x} - \mathbf{x}'\|$$

The likelihood is defined via a sigmoid link function $\sigma(f) = \frac{1}{1+e^{-f}}$, mapping the latent function to class probabilities. Posterior inference is performed using Laplace approximation, where the mode of the posterior is found via Newton-Raphson optimization, and the posterior is approximated as Gaussian around that mode. Hyperparameters, including kernel length scales and output variance, were optimized by maximizing the marginal log-likelihood:

$$\log p(y \mid X) \approx \log p(y \mid \hat{f}) - \frac{1}{2}\log |I + \mathbf{W}^{\frac{1}{2}}\mathbf{K}\mathbf{W}^{\frac{1}{2}}|$$

Note S5. Variational Gaussian Process Classifier (vGPC)

The variational GPC introduces scalable inference via sparse inducing points $\mathbf{Z} = \{\mathbf{z}_m\}_{m=1}^{M}$, which define a lower-dimensional approximation to the full GP. The joint distribution is approximated as:

$$p(f, u) \approx q(f) = \int p(f \mid u) q(u)\, du$$

where $u = f(Z)$, and $q(u) = \mathcal{N}(\mathbf{m}, \mathbf{S})$ is a variational distribution over the inducing variables. The evidence lower bound (ELBO) to be maximized is:

$$\mathcal{L}_{ELBO} = \sum_{i=1}^{N} \mathbb{E}_{q(f_i)}[\log p(y_i \mid f_i)] - KL(q(u) \,||\, p(u))$$

Training was performed using stochastic optimization over mini-batches of data, and the inducing point locations Z were initialized using k-means clustering and updated jointly with kernel parameters.

Note S6. Linear correction model for domain adaption

The correction model is constructed by appending a linear transformation layer to the input of a pre-trained predictive model. For a standardized reaction parameter vector **X**, the transformed input $\mathbf{x}'$ is defined as

$$\mathbf{x}' = \mathbf{W}\mathbf{x} + \mathbf{b},$$

where $\mathbf{W}$ is a diagonal weight matrix and $\mathbf{b}$ is a bias vector.

In this study, the transformation is defined such that

$$\mathbf{x}_{ho\ \ plate\ 1} = \mathbf{W}\mathbf{x}_{hot\ plate\ 2} + \ \boldsymbol{b},$$

which can be equivalently rewritten as

$$\mathbf{x}_{ho\ \ plate\ 2} = (\mathbf{x}_{ho\ \ plate\ 1} - \ \mathbf{b})/\mathbf{W}.$$

Because all reaction parameters are standardized prior to transformation, the parameters $\mathbf{W}$ and b admit a direct numerical interpretation. The diagonal elements of $\mathbf{W}$ determine how changes in $\mathbf{x}_{hot\ plate\ 1}$ are scaled when mapped to $\mathbf{x}_{ho\ \ plate\ 2}$. Smaller values of $\mathbf{W}$ result in larger changes in $\mathbf{x}_{hot\ plate\ 2}$ for a given change in $\mathbf{x}_{hot\ plate\ 1}$, indicating greater sensitivity to hardware-dependent scaling. Conversely, larger values of $\mathbf{W}$ imply reduced sensitivity, as variations in the input are attenuated through the transformation.

The bias term $\mathbf{b}$ represents a systematic offset between the two hardware conditions. A positive bias indicates that the corresponding parameter in $\mathbf{x}_{hot\ plate\ 1}$has a higher baseline value, such that a smaller numerical input is required in $\mathbf{x}_{ho\ \ plate\ 2}$ to achieve the same transformed value. In contrast, a negative bias indicates that a larger numerical input is required in $\mathbf{x}_{hot\ plate\ 2}$ to compensate for the offset.

Overall, the linear correction model provides a simple and transparent numerical framework for quantifying how reaction parameters are rescaled and shifted between different hardware

conditions. When used alongside the transfer-learned predictive model, this formulation enables systematic comparison of hardware-dependent parameter transformations without invoking chemical or mechanistic assumptions.

Supporting References

(1) Berkson, J. Application of the logistic function to bio-assay. *Journal of the American statistical association* 1944, *39* (227), 357-365.

(2) Rumelhart, D. E.; Hinton, G. E.; Williams, R. J. Learning representations by back-propagating errors. *Nature* 1986, *323* (6088), 533-536.

(3) Chen, T. Q.; Guestrin, C. XGBoost: A Scalable Tree Boosting System. *Kdd'16: Proceedings of the 22nd Acm Sigkdd International Conference on Knowledge Discovery and Data Mining* 2016, 785-794. DOI: 10.1145/2939672.2939785.

(4) Turkevich, J.; Stevenson, P. C.; Hillier, J. A study of the nucleation and growth processes in the synthesis of colloidal gold. *Discussions of the faraday society* 1951, *11*, 55-75.

(5) Frens, G. Controlled nucleation for the regulation of the particle size in monodisperse gold suspensions. *Nature physical science* 1973, *241* (105), 20-22.

(6) Haiss, W.; Thanh, N. T.; Aveyard, J.; Fernig, D. G. Determination of size and concentration of gold nanoparticles from UV− Vis spectra. *Analytical chemistry* 2007, *79* (11), 4215-4221.

(7) Panariello, L. Reaction engineering approach to the flow synthesis of nanomaterials for sensing and biomedical applications. UCL (University College London), 2020.

(8) Chavva, S. R.; San Juan, A. M. T.; Jaitpal, S.; Vu, N. N.; Mabbott, S. Efficient production of uniform gold nanoparticles via a streamlined low-cost, semi-automated, open-source platform. *Nanoscale* 2024, *16* (20), 9944-9952.

(9) Sánchez-Iglesias, A.; Pastoriza-Santos, I.; Pérez-Juste, J.; Rodríguez-González, B.; Garcia de Abajo, F. J.; Liz-Marzán, L. M. Synthesis and optical properties of gold nanodecahedra with

size control. *ADVANCED MATERIALS-DEERFIELD BEACH THEN WEINHEIM-* 2006, *18* (19), 2529.

(10) Sun, Y.; Xia, Y. Shape-controlled synthesis of gold and silver nanoparticles. *Science* 2002, *298* (5601), 2176-2179.

(11) Kimling, J.; Maier, M.; Okenve, B.; Kotaidis, V.; Ballot, H.; Plech, A. Turkevich method for gold nanoparticle synthesis revisited. *The Journal of Physical Chemistry B* 2006, *110* (32), 15700-15707.

(12) Nocedal, J. Updating quasi-Newton matrices with limited storage. *Mathematics of computation* 1980, *35* (151), 773-782.
